# Genome-wide identification and functional analysis of oleosin genes in *Brassica napus* L.

**DOI:** 10.1186/s12870-019-1891-y

**Published:** 2019-07-04

**Authors:** Kang Chen, Yongtai Yin, Si Liu, Zhenyi Guo, Kai Zhang, Yu Liang, Lina Zhang, Weiguo Zhao, Hongbo Chao, Maoteng Li

**Affiliations:** 1 Department of Biotechnology, College of Life Science and Technology, Huazhong University of Science and Technology, Wuhan, 430074 China; 2 Hubei Collaborative Innovation Center for the Characteristic Resources Exploitation of Dabie Mountains, Huanggang Normal University, Huanggang, China

**Keywords:** *Brassica napus*, Oleosin, Gene evolution, Fatty acid, Oil content

## Abstract

**Background:**

Rapeseed is the third largest oil seed crop in the world. The seeds of this plant store lipids in oil bodies, and oleosin is the most important structural protein in oil bodies. However, the function of oleosin in oil crops has received little attention.

**Results:**

In the present study, 48 oleosin sequences from the *Brassica napus* genome were identified and divided into four lineages (T, U, SH, SL). Synteny analysis revealed that most of the oleosin genes were conserved, and all of these genes experienced purifying selection during evolution. Three and four important oleosin genes from *Arabidopsis* and *B. napus,* respectively*,* were cloned and analyzed for function in *Arabidopsis*. Overexpression of these oleosin genes in *Arabidopsis* increased the seed oil content slightly, except for *BnaOLE3*. Further analysis revealed that the average oil body size of the transgenic seeds was slightly larger than that of the wild type (WT), except for *BnaOLE1*. The fatty acid profiles showed that the linoleic acid content (13.3% at most) increased and the peanut acid content (11% at most) decreased in the transgenic lines. In addition, the seed size and thousand-seed weight (TSW) also increased in the transgenic lines, which could lead to increased total lipid production.

**Conclusion:**

We identified oleosin genes in the *B. napus* genome, and overexpression of oleosin in *Arabidopsis* seeds increased the seed weight and linoleic acid content (13.3% at most).

**Electronic supplementary material:**

The online version of this article (10.1186/s12870-019-1891-y) contains supplementary material, which is available to authorized users.

## Background

*Brassica napus*, which is closely related to the model plant *Arabidopsis*, is one of the most important oil crops, providing approximately 15% of the vegetable oil worldwide [1, 2]. *B. napus* shares a highly conserved genome sequence with *Arabidopsis*, especially for genes associated with lipid biosynthesis, which has been extensively studied [3, 4]. However, detailed analysis of the lipid metabolism pathway is required to understand homology gene function differentiation in this complex genome. Identification and functional analysis of homologous genes in *B. napus* has become easier with the release of the *Arabidopsis* and *B. napus* genomes.

Lipids are the main reserves in *B. napus* seeds and account for 35–50% of the dry weight. Triacylglycerol (TAG) is the main component of lipids and is stored in oil bodies [1, 5]. The oil body, an organelle that is widely distributed in lipid storage cells, is approximately 0.5 to 2 μm in diameter and contains a liquid matrix of neutral lipids surrounded by a hemi-unit membrane with structural proteins. The oil body serves as a natural protective system against fatty acid oxidation and maintains lipid stability under extreme stress conditions [6]. A well-known hypothesis is that the oil body is synthesized in the ER and then released to the cytoplasm via a budding mechanism [7, 8]. Although oil bodies can remain stable and do not aggregate or coalesce even after long-term storage when the environment remains unchanged, such as in mature seed cells or in vitro [9], the structure of this organelle does not remain constant during seed development, and the size of the oil body changes during seed maturation. The size of the oil body first increases and then decreases during seed development [10].

Three main proteins (oleosin, caleosin and steroleosins) are inserted in the phospholipid membrane [11, 12]. In addition, several putative adipose-regulatory proteins (such as *SEIPIN*), oil body-associated protein 1 (*OBAP1*) and lipid droplet-associated proteins were also reported to be present in the oil body [5, 13, 14]. Oleosin, which is synthesized in the ER, accounts for 80–90% of the oil body structure proteins and plays an important role in lipid storage [11]. Vance et al. [15] first identified the oleosin sequence from maize and proposed that oleosin may serve as the recognition signal for the specific binding of lipase to lipid bodies in the lipid degradation pathway [15, 16]. The oleosins from sesame, Pinus and *B. napus* were also cloned [17–19]. The oleosin peptide can be divided into three parts: a short amphipathic N-terminal sequence, a central hydrophobic domain and an amphipathic C-terminal sequence [5, 16, 20]. Thirty amino acids of the C-terminal sequence near the central domain can form an α-helical structure, which interacts horizontally with the PL layer on the oil body surface. The N- and C-terminal peptides present outside the oil body might be the target of lipase for oil body degradation or other metabolic pathways [7, 21]. The central hydrophobic domain, composed of 72 of the most highly conserved amino acids, is possibly one of the hallmarks of the oil body protein because this region has not been identified in the structural proteins of other lipid storage organelles [5, 21]. The conserved 72 amino acids can form a hairpin structure that is inserted into the phospholipid membrane, in which a highly conserved Pro knot (PX5SPX3P) forms the loop of the hairpin structure. The three Pro residues and one Ser residue are present in all oleosins from diverse plant species and green algae without any substitution, and substitution of the conserved Pro residues leads to abnormal localization [22]. Oleosins from diverse species have been reported, all of which can be divided into six lineages (P, U, SL, SH, T and M) [5, 16]. Among the six lineages, the M lineage is present in *Lauraceae*, and the T lineage was detected in only the tapeta of *Brassicaceae* [16, 20, 23]. The P lineage was mainly distributed in green algae and might be the origin of U oleosin, which further gave rise to the SL and SH lineages [5].

Many studies have proven that adjustment of the oleosin protein level could prevent fusion of the oil body and maintain oil body size during seed development [24–26]. The size of the oil droplets in wild-type (WT) and *AtOLE1*-knockout *Arabidopsis* seeds was uniform in the early stages of seed development [10]. The lack of oleosins causes the oil body to compress and fuse, resulting in an enlarged oil body in the middle stage of seed development [11, 27, 28]. Several homologous oleosin proteins from *Arabidopsis thaliana* were studied, and *OLE1* and *OLE4* were found to negatively regulate oil body size, whereas *OLE2* might increase the oil body size [10]. In a OLE-knockout *Arabidopsis* mutant, although the oil body shape was irregular, the total fatty acid content did not change, except for the levels of eicosenoic acid (C20:1) and oleic acid (C18:1). The plants were also sensitive to low temperature during germination [6]. Similar results could be proved by heterologous expression of castor bean oleosin in *Arabidopsis*, which led to a 20% increase in the ricinoleic acid content in TAGs [29]. On the other hand, coexpression of oleosins with other genes in the TAG biosynthesis pathway could enhance the oil content [12], which might represent a new role of oleosins in the development of genetically modified crops. *OLE* also plays a role in *Arabidopsis* tapetosome formation and pollen development [30]. As a lipid storage structure, the quantity and size of oil bodies can reflect the oil content of seeds to some extent. Although there is some controversy regarding the relationship of oil body number and size with oil content [31, 32], high oil levels tend to lead to higher oil body production or sizes than low oil level [31], and the high oil content might be associated with increased oleosin gene copy numbers and increased gene expression levels.

However, the function of oleosins from important oil corps has received little attention. In the present study, all of the oleosin sequences from the *B. napus* genome were identified, and their structures, evolution and synteny relationship with oleosins from other Cruciferae plants were analyzed. In addition, 7 important oleosin genes from *Arabidopsis* and *B. napus* were cloned and transformed into *Arabidopsis* for functional analysis. The present results revealed that overexpression of oleosin in *Arabidopsis* could affect oil body size as well as seed size and seed weight, so oil production could be increased. In addition, transgenic seeds also show decreased freezing tolerance. This study provides a foundation for future studies regarding the oleosin superfamily in *B. napus.*

## Results

### Genome-wide identification of oleosin family genes in the genome of *B. napus*

Based on the gene sequences of 17 oleosin genes obtained from the Arabidopsis Information Resource (TAIR) using oleosin as the query, the oleosin genes from the *B. napus* genome were searched using BLAST in the CNS-Genoscope database. In total, 65 genes that share high similarity with *Arabidopsis* oleosin genes were identified, and 48 genes containing the oleosin family domain PF01277 in the Pfam database were selected for further synteny analysis (Table 1). In addition, 30 and 28 oleosin genes were identified from *Brassica rapa* and *Brassica oleracea*, respectively (Additional file 5: Table S1). The physicochemical parameters of each oleosin protein were calculated by ExPASy, and a considerable difference in these parameters was observed, which might result from the highly variable terminal sequences in these oleosin genes. The results revealed that most of the oleosin proteins had molecular masses < 25 kDa, except *BnaOLE22*, *BnaOLE23*, *BnaOLE32, BnaOLE35* and *BnaOLE37*. Forty-three of these proteins have relatively high isoelectric points (pI> 7). Nearly all of these proteins have a high aliphatic index, and the GRAVY value of these proteins is close to zero, which shows that these oleosin proteins exhibit amphipathy.

**Table 1:** *OLE* genes in *B. napus* genome and their sequence characteristics and physicochemical parameters prediction

Gene ID	Name	family	chr.	gene position		gene length	exon	intron	Number of aa	Mol.Wt.	Theoretical pI	Aliphatic index	GRAVY
				start	end								
BnaA01g14480D	BnaOLE1A01	SH	A01	7,305,082	7,306,307	1226	3	2	170	18,556.14	9.52	86.65	−0.194
BnaA01g26680D	BnaOLE8A01	U	A01	18,669,237	18,669,731	495	1	0	164	18,141.98	9.39	96.4	0.232
BnaA02g29000D	BnaOLE3	SL	A02	21,253,735	21,254,824	1090	2	1	188	20,001.84	9.15	97.02	−0.005
BnaA02g35480D	BnaOLE18	U	A02_R	273,275	273,804	530	1	0	131	13,569.19	8.8	121.15	0.926
BnaA03g02100D	BnaOLE19	T	A03	962,155	963,313	1159	2	1	272	27,116.61	10.21	77.43	0.057
BnaA03g02110D	BnaOLE20	T	A03	964,861	966,041	1181	2	1	207	21,751.38	10	82.46	−0.189
BnaA03g02120D	BnaOLE13A03	T	A03	967,459	968,374	916	1	0	79	7931.6	5.75	136.08	1.597
BnaA03g02140D	BnaOLE21	T	A03	975,911	977,658	1748	3	2	119	12,160.68	10.3	115.63	0.759
BnaA03g27410D	BnaOLE22	SL	A03	13,512,809	13,516,176	3368	5	4	778	86,260.73	7.3	93.07	−0.03
BnaA03g34830D	BnaOLE8A03	U	A03	17,011,078	17,011,575	498	1	0	165	17,821.37	6.38	95.76	0.421
BnaA03g40390D	BnaOLE17A03	T	A03	20,190,729	20,192,198	1470	3	2	251	27,929.33	9.08	92.11	−0.162
BnaA03g47170D	BnaOLE1A03	SH	A03	24,188,857	24,190,194	1338	2	1	183	20,001.69	9.3	84.81	−0.234
BnaA04g15210D	BnaOLE6A04	SH	A04	12,660,472	12,660,912	441	1	0	146	15,657.25	8.66	92.88	0.277
BnaOLE2	BnaOLE2	U	A04	9,050,747	9,051,738	992	1	0	220	23,048.11	9.1	77.59	−0.083
BnaA05g21590D	BnaOLE8A05	U	A05	16,610,133	16,610,630	498	1	0	165	17,932.6	9.52	97.52	0.339
BnaA06g03370D	BnaOLE23	SL	A06	2,059,111	2,061,705	2595	7	6	517	57,648.71	8.59	92.75	−0.032
BnaA07g14960D	BnaOLE2A07	SH	A07	13,061,213	13,062,594	1382	3	2	200	21,284.38	9.3	88.3	−0.046
BnaA08g14540D	BnaOLE1	T	A08	12,282,608	12,283,829	1222	2	1	180	19,514.98	9.15	84.61	−0.248
BnaA08g22860D	BnaOLE24	SL	A08	16,473,505	16,474,574	1070	2	1	209	20,340.83	9.7	99.43	0.75
BnaA09g02110D	BnaOLE4A09	U	A09	1,040,084	1,041,270	1187	2	1	188	19,879.71	6.91	96.97	0.045
BnaA10g05240D	BnaOLE25	SH	A10	2,963,139	2,963,633	495	1	0	164	17,864.6	9.1	98.17	0.347
BnaA10g05760D	BnaOLE3A10	T	A10	3,966,784	3,967,565	782	2	1	149	15,574.98	10.1	104.09	0.286
BnaA10g23950D	BnaOLE26	T	A10	15,705,746	15,706,808	1063	2	1	175	17,006.96	10.12	110.51	0.852
BnaA10g23960D	BnaOLE27	T	A10	15,711,917	15,713,045	1129	2	1	195	19,523.32	10.91	119.33	0.841
BnaA10g30180D	BnaOLE28	T	A10_R	2,101,896	2,103,711	1816	2	1	204	20,585.57	9.4	78.04	−0.185
BnaA10g30190D	BnaOLE29	SH	A10_R	2,105,832	2,106,825	994	2	1	108	11,032.19	11.17	116.57	0.807
BnaC01g17050D	BnaOLE1C01	U	C01	11,676,131	11,677,343	1213	3	2	170	18,571.08	9.39	82.65	−0.224
BnaC01g34070D	BnaOLE8C01	T	C01	33,257,398	33,257,892	495	1	0	164	17,864.6	9.1	98.17	0.347
BnaC02g01550D	BnaOLE30	T	C02	627,160	628,665	1506	2	1	149	14,826.83	11.28	107.65	0.682
BnaC02g01560D	BnaOLE31	T	C02	629,668	630,975	1308	2	1	157	15,236.32	10.72	109.87	0.73
BnaC02g01570D	BnaOLE32	SL	C02	633,723	637,275	3553	3	2	870	88,165.02	10.4	51.98	−0.891
BnaC02g37030D	BnaOLE4C02	T	C02	40,002,669	40,003,899	1231	2	1	191	20,248.17	7.89	101.1	0.058
BnaC03g03140D	BnaOLE33	T	C03	1,516,588	1,518,583	1996	3	2	214	22,584.38	10.17	81.59	−0.196
BnaC03g03150D	BnaOLE13C03	T	C03	1,519,876	1,520,984	1109	2	1	116	12,072.39	9.3	106.98	0.456
BnaC03g03170D	BnaOLE34	SL	C03	1,525,165	1,526,940	1776	2	1	120	12,506.14	9.9	133.42	1.066
BnaC03g32430D	BnOLE37	U	C03	19,921,970	19,925,218	3249	4	3	777	86,175.33	6.29	93.56	−0.004
BnaC03g40520D	BnaOLE8C03	SL	C03	25,478,827	25,479,324	498	1	0	165	17,778.43	6.9	101.7	0.474
BnaC04g32530D	BnaOLE2C04	SH	C04	34,250,286	34,251,730	1445	2	1	229	24,575.09	9.34	75.02	−0.111
BnaC04g38160D	BnaOLE6C04	U	C04	39,433,420	39,433,857	438	1	0	145	15,567.15	7.85	98.21	0.334
BnaC05g34600D	BnaOLE8C05	U	C05	33,883,325	33,883,822	498	1	0	165	17,994.67	9.52	96.3	0.352
BnaC06g02430D	BnaOLE35	SL	C06	3,184,669	3,187,278	2610	7	6	517	57,886.95	8.78	92.17	−0.05
BnaC06g12930D	BnaOLE2C06	T	C06	15,634,774	15,636,282	1509	3	2	200	21,275.37	9.3	88.3	−0.048
BnaC07g31360D	BnaOLE17C07	SH	C07	35,479,311	35,480,772	1462	3	2	251	27,859.39	9.22	94.82	−0.139
BnaC07g39370D	BnaOLE1C07	U	C07	40,278,874	40,279,959	1086	2	1	186	20,565.22	9.15	82.9	−0.354
BnaC08g03140D	BnaOLE5	SH	C08	2,826,379	2,826,801	423	1	0	140	15,613.35	10.16	108.71	0.513
BnaC08g11970D	BnaOLE1C08	SH	C08	17,257,794	17,259,450	1657	3	2	179	19,424.86	9.15	85.08	−0.288
BnaC09g27370D	BnaOLE3C09	T	C09	29,151,518	29,153,074	1557	2	1	149	15,711.18	9.99	104.09	0.311
BnaC09g47930D	BnaOLE36	SL	C09	47,002,663	47,003,720	1058	2	1	171	16,666.58	10.39	107.37	0.839

### Sequence and phylogenetic analyses of the OLE genes

**Fig. 1:**
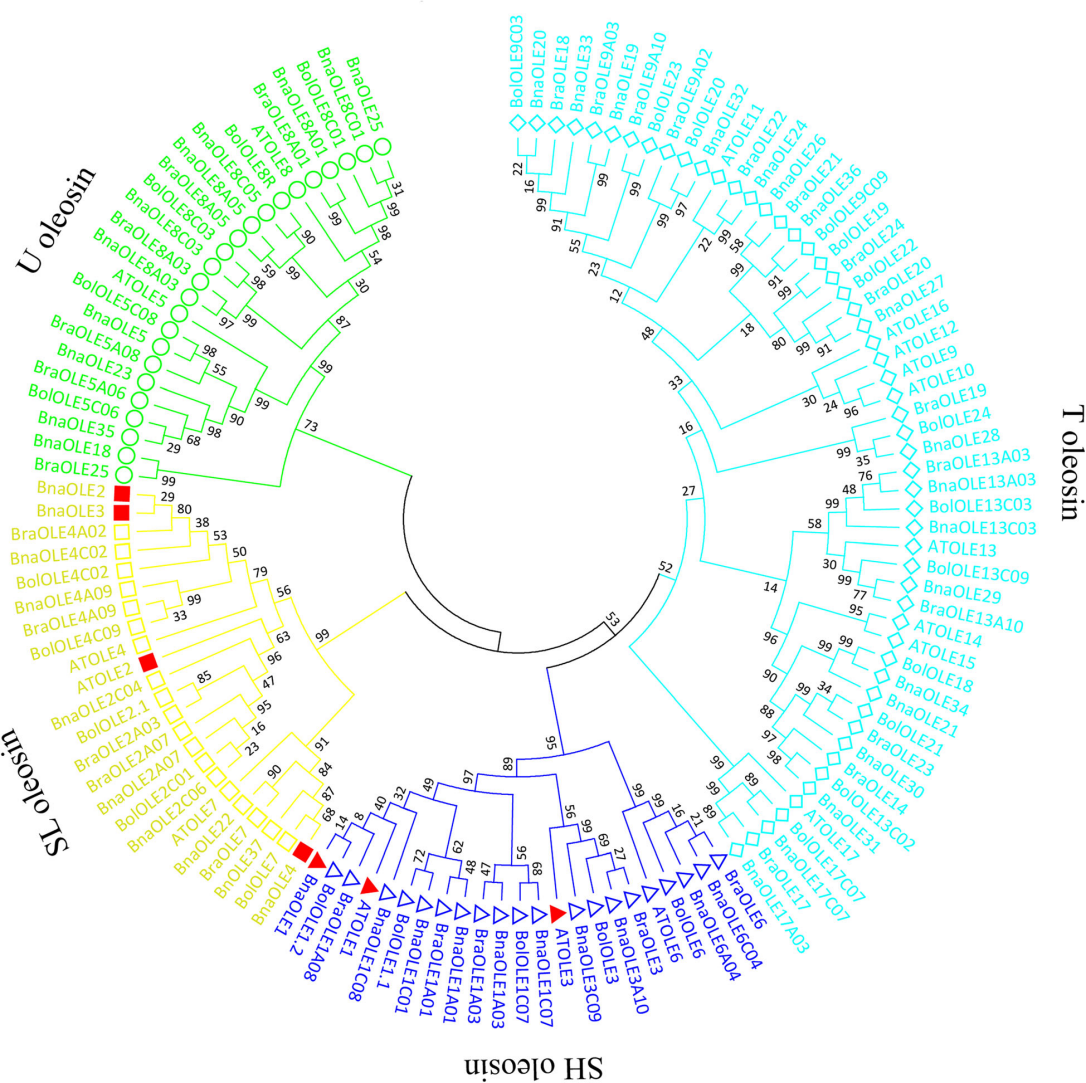
Phylogenetic analysis of the *A. thaliana, B. rapa, B. oleracea*, and *B. napus* oleosin genes. The conserved central amino acid sequences of the OLE proteins were used to construct the cladogram using ClustalX and MEGA6. Different lineages of oleosins are colored differently, and the solid red graphics represent the gene that we selected for further functional analysis

Sequence alignment was performed to analyze the similarity of oleosin genes between *B. napus* and other *Brassica* species using ClustalW. It was revealed that all of these oleosins contained a 72-amino-acid conserved sequence, exhibiting relatively high conservation. The conserved 72-residue sequence was submitted to Multiple Expectation Maximization for Motif Elicitation (MEME) for motif structure analysis, which indicated that the most well conserved sequence was the Pro loop (PX5SPX3P), and most of the X residues were the nonpolar Ile, Phe, Leu and Val in *B. napus*. *BnaOLE17C07* and *BnaOLE17A03* were different from the other oleosin genes; the third prolines of these proteins had been replaced by Thr (a neutral, nonpolar amino acid) (Additional file 1: Figure S1). The conserved 72-residue sequence was analyzed by phylogenetic analysis, revealing that the 48 oleosins of *B. napus* could be divided into the T, U, SL and SH lineages (Fig. 1, Additional file 2: Figure S2). Further analysis showed that T oleosin is the largest clade, including over 1/3 of the oleosins in *B. napus*, and that the other 3 lineages had similar members. The genes from the A genome and C genome of *B. napus* always appeared in pairs in most of the clades. The corresponding genes in their ancestors *B. rapa* and *B. oleracea* were also closely related, which indicated that these genes evolved from the same ancestor. Although syntenic genes were not identified for some *Bna*OLEs, we could speculate on the evolutionary relationships of these genes. For instance, *Bna*OLE5 was clustered in the same clade as *Bna*OLE8C01 and showed a close phylogenetic relationship with AtOLE5, which may indicate the evolutionary history of these genes.

**Fig. 2:**
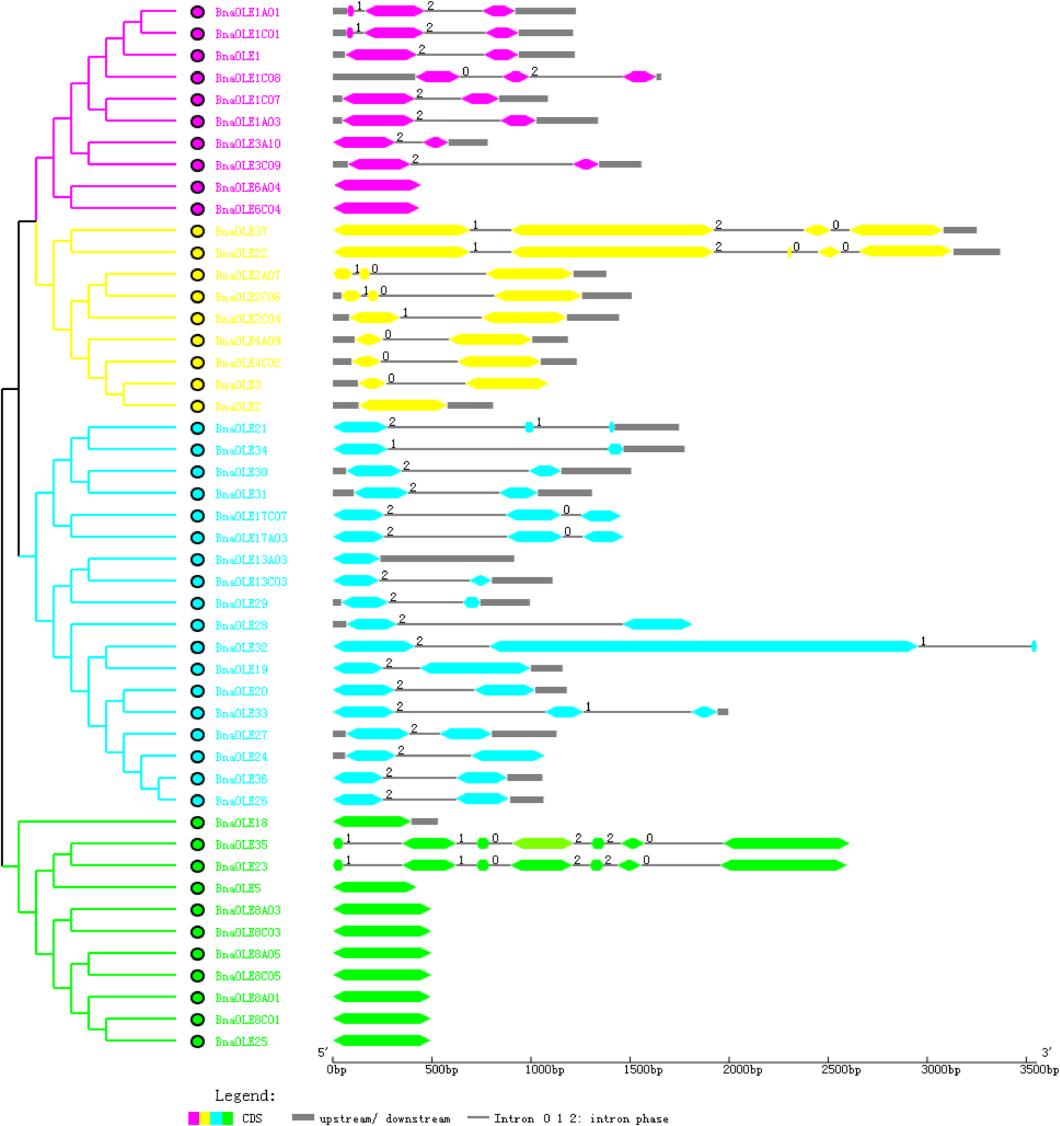
Genetic structure of *B. napus* oleosin genes. Different lineages of oleosins are colored differently. Green, yellow, dark blue and sky blue represent U, SL, SH and T oleosins, respectively. Round double-sided wedge boxes represent exons; black lines represent introns; gray boxes represent untranslated regions (UTRs); and the numbers near the introns indicate the intron phase. If an intron is between two complete codons, then the intron phase is defined as 0; if an intron is after the first or second nucleotide within the codon, the intron phase is defined as phase 1 or phase 2

The lengths of most oleosin genes from *B. napus* were shorter than 2 kb, except for *BnaOLE22*, *BnaOLE23*, *BnaOLE32*, *BnaOLE35* and *BnaOLE37*. These five genes did not belong to the same lineages and shared low sequence identity with each other in addition to the same lineages. Each clade of *B. napus* oleosins contains different intron-exon structural features (Fig. 2). In general, T oleosins possess more exons than the other three types of oleosins. Most U oleosins have no introns, except for *BnaOLE23* and *BnaOLE35*, which are composed of many more amino acid residues than the other U oleosins. In addition to the other three long-chain oleosin genes, these genes also contain more exons than other genes from the same lineages. However, *BnaOLE23* and *BnaOLE35* show the same genetic structure as *BnaOLE22* and *BnaOLE37*, so the gene pairs from the A genome and C genome have the same gene structure. Most of the oleosins from the other three clades contains two or three exons, with some exceptions that have no intron. For example, the SL oleosins *BnaOLE22* and *BnaOLE37* and the T oleosin *BnaOLE32* possess more introns than the oleosins from the same lineages. Oleosins with a large number of introns often encode more amino acids than other oleosins. However, the SH oleosin genes *BnaOLE6A04* and *BnaOLE6C04*, which have similar sequence lengths as other SH oleosin genes, have no introns. Despite the intron structure and sequence difference, all of the introns in oleosins are U2-type splice GT-AG introns [33, 34].

### Chromosomal location, duplication patterns and synteny analysis of the oleosin genes in *B. napus*

**Fig. 3:**
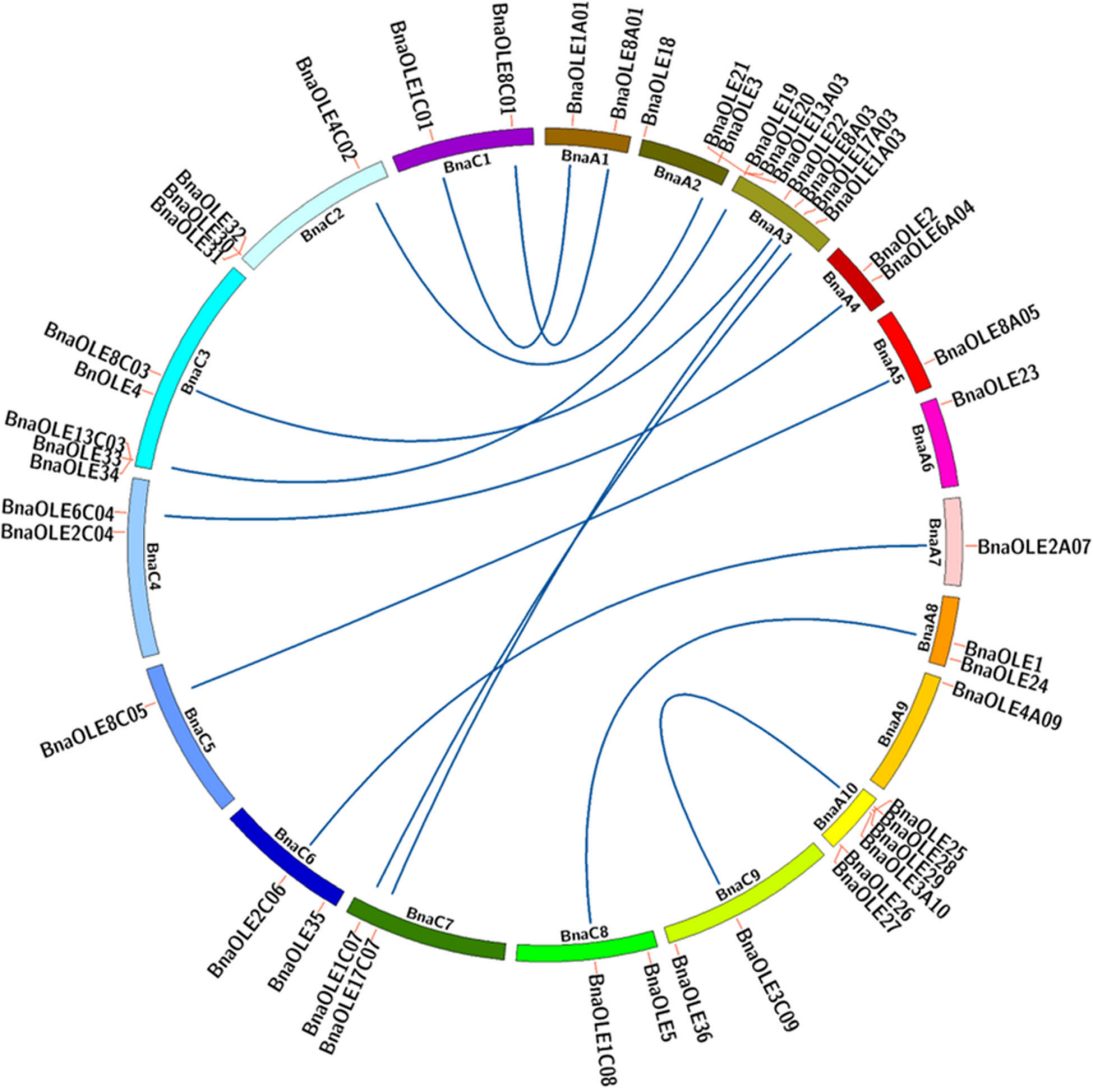
Distribution of the oleosin gene in the *B. napus* genome and the syntenic relationship between oleosin genes. The chromosomal location information for the oleosin genes was obtained from the CNS-Genoscope database. The dark blue line indicates that these two genes located in the A genome and C genome are syntenic genes

The chromosomal positions of the *BnaOLE* gene were analyzed, and most of the chromosomes possessed one or two oleosin genes except for the A03, A10, C02 and C03 chromosomes, and 8, 6, 4 and 5 oleosin genes were observed in these four chromosomes, respectively (Fig. 3). In addition, gene clusters mainly composed of T oleosins were found in some chromosomes; for instance, four genes encoding T oleosins, namely, *BnaOLE19*, *BnaOLE20*, *BnaOLE13A03* and *BnaOLE21,* were located in clusters on the A03 chromosome, one of the reasons for which might be that nine T oleosins were clustered on *A. thaliana* chromosome 5. Most of the genes from the A genome appear in pairs with genes from the C genome. Intriguingly, two *BnaOLE* genes located on chromosome A3 were closely linked to paralogous genes located on chromosome C7, which might result from segmental duplication and chromosomal rearrangement during the long evolutionary history. The *Brassica* species experienced several whole-genome triplication (WGT) events, which contributed to gene-level evolution [35]^.^ Gene duplication events were investigated to understand the genome expansion mechanism of the *BnaOLE* gene family in *B. napus*. Twelve tandemly duplicated genes (*BnaOLE19*/*BnaOLE20*/*BnaOLE13A03*/*BnaOLE21*, *BnaOLE26*/*BnaOLE27*, *BnaOLE30*/*BnaOLE31*/*BnaOLE32* and *BnaOLE33*/*BnaOLE13C03*/*BnaOLE34*) located on chromosomes A03, A10, C02 and C03 were identified, and all twelve oleosins belonged to T oleosins.

**Fig. 4:**
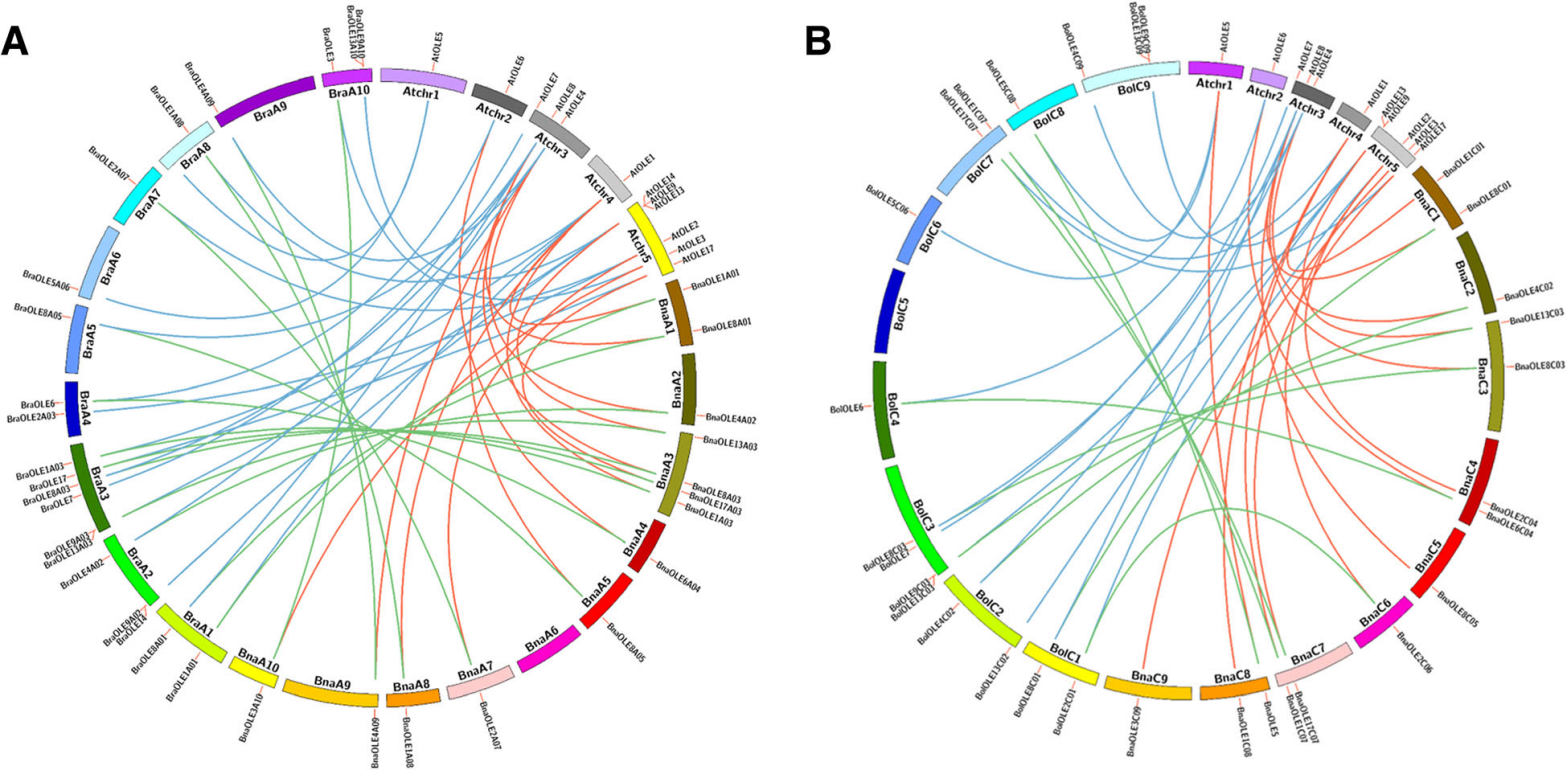
Synteny analysis map of oleosin genes. **a** Synteny analysis map of oleosin genes in the A genome in *A. thaliana*, *B. rapa* and *B. napus* chromosomes. The red lines indicate that these two genes from *A. thaliana* and *B. napus* are syntenic genes. The blue lines indicate that these two genes from *A. thaliana* and *B. rapa* are syntenic genes. The green lines indicate that these two genes from *B. rapa* and *B. napus* are syntenic genes. **b** Synteny analysis map of oleosin genes in the C genome in *A. thaliana*, *B. oleracea* and *B. napus* chromosomes. The red lines indicate that these two genes from *A. thaliana* and *B. napus* are syntenic genes. The blue lines indicate that these two genes from *A. thaliana* and *B. oleracea* are syntenic genes. The green lines indicate that these two genes from *B. oleracea* and *B. napus* are syntenic genes

The *Brassicaceae* genome has experienced complex genome duplication and rearrangement [36]. We also analyzed the synteny maps of the oleosin genes in the *B. napus* genome and the homologous genes in *A. thaliana*, *B. rapa* and *B. oleracea* (Fig. 4)*.* Gene duplication and gene loss were also observed in the *OLE* gene family. Except for some genes, such as *AtOLE7,* we did not identify syntenic genes. We found that most *AtOLE* genes had syntenic relationships with two or more *BraOLE* and *BolOLE* genes as a result of gene duplication, and these duplicated OLEs originating from the same *AtOLE*s in *B. rapa* and *B. oleracea* were distributed on different chromosomes. In addition, *BraOLE*s always appear in pairs with *BolOLE*s, and nearly all of these homologous *BraOLE* or *BolOLE* genes maintained a syntenic relationship with *BnaOLEs*. Given the close genetic relationship between *B. rapa*, *B. oleracea* and *B. napus*, a low level of gene loss was also observed in *B. napus*; for example, *At*OLE1 had syntenic relationships with *BraOLE1A01*, *BraOLE1A03* and *BraOLE1A08*, and syntenic genes of these three genes could be detected in the *B. napus* genome. In addition, we did not detect syntenic relationships of *AtOLE10* in the A genome.

Synonymous and nonsynonymous values were examined to assess the selective pressure on duplicated *BnaOLE* genes (Table 2). The largest *Ka/Ks* ratio of the gene pairs between *B. napus* and *A. thaliana* was 0.53, and the two largest *Ka/Ks* ratios were associated with *AtOLE13*. In contrast, the *Ka/Ks* ratio of *AtOLE1* and the related orthologous OLE gene was the lowest. Different genes experience various degrees of selective pressure, and OLE1 is more highly conserved than other OLE genes, implying that OLE1 might perform more important functions than the other genes. In addition, the *Ka/Ks* ratio of T oleosins, such as *AtOLE13* and *AtOLE17,* was relatively higher than that of the other oleosin lineages, which might be due to the high numbers of T oleosins.

**Table 2:** Non-synonymous (Ka) and synonymous (Ks) nucleotide substitution rates for *A. thaliana* and *B. napus*ole gene

*A. thaliana* ID	*A. thaliana* gene	*B. napus* ID	*B. napus* gene	Ka	Ks	Ka/Ks
AT1G48990	*At*OLE5	*Bna*C08g03140D	*Bna*OLE5	0.1408	0.61476	0.229032
AT2G25890	*At*OLE6	*Bna*A04g15210D	*Bna*OLE6A04	0.08926	0.53933	0.165502
		*Bna*C04g38160D	*Bna*OLE6C04	0.10466	0.54036	0.193686
AT3G18570	*At*OLE8	*Bna*A05g21590D	*Bna*OLE8A05	0.10166	0.46093	0.220554
		*Bna*A01g26680D	*Bna*OLE8A01	0.15212	0.41	0.371024
		*Bna*A03g34830D	*Bna*OLE8A03	0.11772	0.4827	0.243878
		*Bna*C05g34600D	*Bna*OLE8C05	0.09823	0.40958	0.239831
		*Bna*C01g34070D	*Bna*OLE8C01	0.13442	0.37374	0.359662
		*Bna*C03g40520D	*Bna*OLE8C03	0.12162	0.49017	0.248118
AT3G27660	*At*OLE4	*Bna*A02g29000D	*Bna*OLE4A02	0.08985	0.43793	0.20517
		*Bna*A09g02110D	*Bna*OLE4A09	0.10473	0.3736	0.280327
		*Bna*C02g37030D	*Bna*OLE4C02	0.10194	0.43748	0.233016
AT4G25140	*At*OLE1	*Bna*A01g14480D	*Bna*OLE1A01	0.05086	0.53381	0.095277
		*Bna*A03g47170D	*Bna*OLE1A03	0.06531	0.53037	0.12314
		*Bna*A08g14540D	*Bna*OLE1A08	0.05022	0.48812	0.102885
		*Bna*C01g17050D	*Bna*OLE1C01	0.04063	0.57389	0.070798
		*Bna*C07g39370D	*Bna*OLE1C07	0.08101	0.5862	0.138195
		*Bna*C08g11970D	*Bna*OLE1C08	0.05527	0.47217	0.117055
AT5G07550	*At*OLE13	*Bna*A03g02120D	*Bna*OLE13A03	0.18617	0.35046	0.531216
		*Bna*C03g03150D	*Bna*OLE13C03	0.17112	0.37186	0.460173
AT5G40420	*At*OLE2	*Bna*A07g14960D	*Bna*OLE2A07	0.08676	0.46651	0.185977
		*Bna*C04g32530D	*Bna*OLE2C04	0.1523	0.56784	0.268209
		*Bna*C06g12930D	*Bna*OLE2C06	0.08442	0.4767	0.177093
AT5G51210	*At*OLE3	*Bna*A10g05760D	*Bna*OLE3A10	0.08088	0.43697	0.185093
		*Bna*C09g27370D	*Bna*OLE3C09	0.08129	0.47551	0.170953
AT5G61610	*At*OLE17	*Bna*A03g40390D	*Bna*OLE17A03	0.24568	0.43828	0.560555

### Gene cloning of AtOLEs and subcellular localization of oleosin proteins

Previous research has shown that the ortholog of *OLE1* and *OLE2* is located in the region of the quantitative trait locus (QTL) of seed oil content [37], which is evidence that oleosin might contribute to TAG accumulation. Therefore, we cloned three oleosin genes from *A. thaliana* and four of the homologous genes from *B. napus* to verify the effect of these genes on seed oil content in *Arabidopsis. AtOLE1*, *AtOLE2* and *AtOLE3* possess 173, 199 and 141 amino acids, respectively, and *BnaOLE*s possess relatively high numbers of amino acids. *BnaOLE1*, *BnaOLE2*, *BnaOLE3* and *BnaOLE4* possess 180, 220, 188 and 193 amino acids, respectively.

**Fig. 5:**
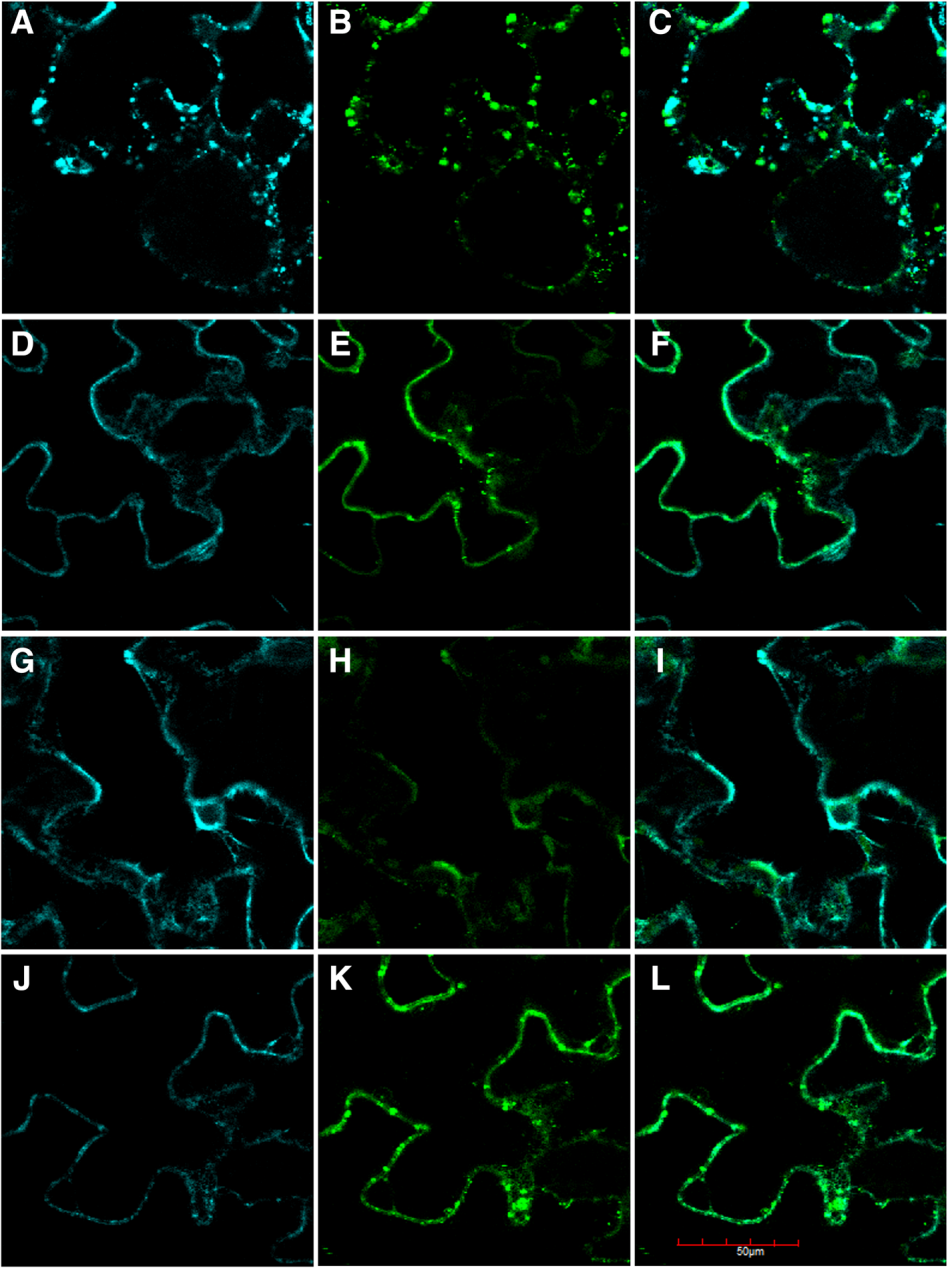
Subcellular localization of BnaOLEs. **a**, **b**, and **c** represent the subcellular location of BnaOLE1; **d**, **e** and **f** represent the subcellular location of BnaOLE2; **g**, **h** and **i** represent the subcellular location of BnaOLE3; and **j**, **k** and **l** represent the subcellular location of BnaOLE4. **a**, **d**, **g**, **j**: plasma membrane-CFP marker; **b**, **e**, **h**, **k**: EGFP fluorescence; **c**, **f**, **i**, **l**: merged fluorescence of EGFP and plasma membrane-CFP marker. Scale bar: 50 μm

The subcellular localization of oleosins from *B. napus* was analyzed, and the vector pCambia1303-EGFP-DsRed3 containing the target protein was constructed, in which the fluorescent protein was attached to the oleosin protein. Therefore, we could check the oleosin distribution by monitoring the fluorescence signal in cells. The constructed plasmids were transformed into tobacco leaves for transient expression with Agrobacterium GV3101. The results revealed that the cellular localization of BnaOLE*s* partially overlapped with the cell membrane marker (Fig. 5). The results for AtOLE*s* were similar, and the fluorescence signals for these proteins coincided with the cell membrane and partially overlapped with the chloroplast (Additional file 3: Figure S3). This result indicated that the oleosin protein may be located in the cell membrane before oil body formation.

### Overexpression of OLEs in *Arabidopsis* could affect the fatty acid content

**Fig. 6:**
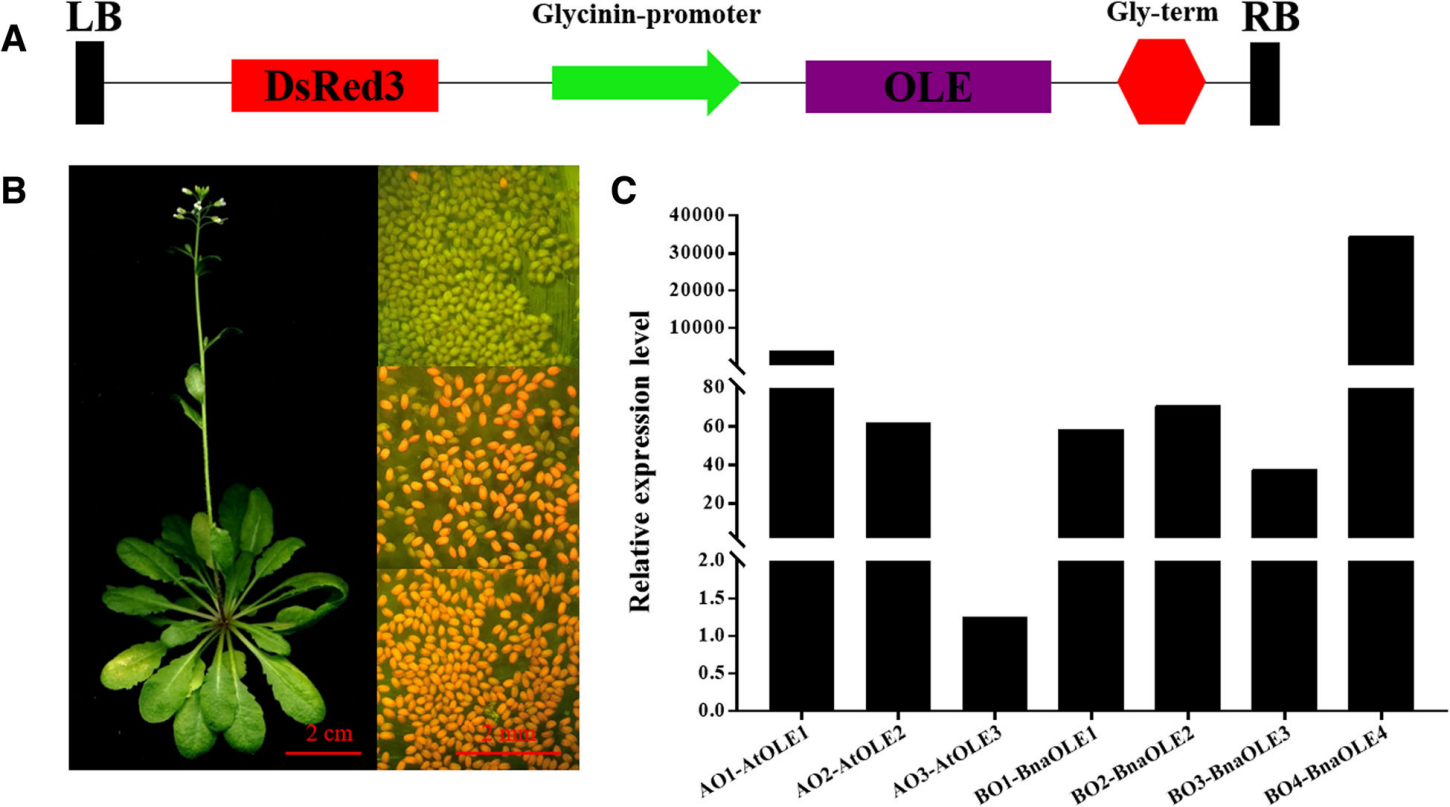
Plant transformation and selection of transgenic seeds. **a** Schematic representation of the plant expression vector used for plant transformation. **b** Different generations of transgenic plants under green light. The red seeds and siliques represent the transgenic lines, while the green represents the WT. **c** Relative expression of target genes in the siliques of the T3 generation of transgenic plants. The expression of *AtOLE3* in WT was assigned as 1

The plant expression vector with the glycinin promoter was constructed and then transferred into *Arabidopsis* to generate the *OLE* overexpression lines. DsRED, a type of fluorescent protein, was also added to the vector (Fig. 6). It was easy to identify the transgenic seeds by observing the red fluorescence through a filter. This approach is more convenient than the selection of transgenic plants based on antibiotic resistance. The transgenic lines overexpressing *OLE*s showed no visible morphological variation compared with the WT. To investigate the influence of overexpression of these genes on lipid metabolism, homozygous transgenic T3 lines were used to analyze the oil content and fatty acid profiles using gas chromatography.

**Fig. 7:**
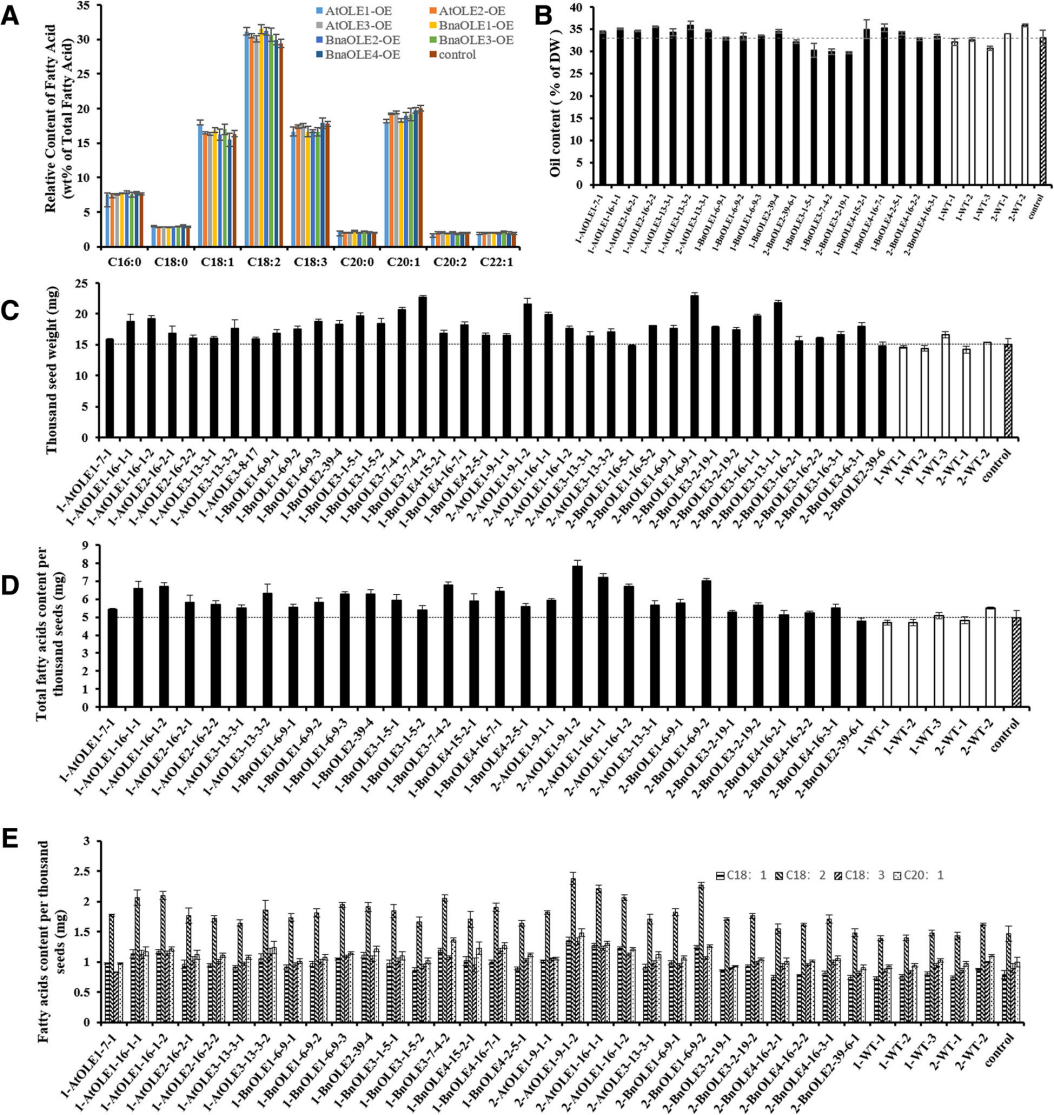
Oil content and fatty acids profiles in *Arabidopsis* seeds. **a** Relative levels of fatty acids in transgenic seeds and the WT; (**b**) oil content in transgenic seeds and the WT; (**c**) thousand-seed weight of transgenic plants and the WT; (**d**) total fatty acid weight per thousand seeds of transgenic plants and the WT; (**e**) levels of different types of fatty acids per thousand seeds of transgenic plants and the WT. At least three replicates were used for each sample. T-test was used to measure significance differences between lines and the transgenic lines showed significant difference with the control

It was revealed that the oil content of transgenic lines showed different variation compared with the WT (Fig. 7a, b, Table 3). The seed oil content of *OLE*-OE in different lines varied from 29.2 to 35.6%. Compared with the WT (with an oil content of 32.3%), the oil content in most of the transgenic lines increased slightly, except in *BnaOLE3*-OE. Further analysis showed that the oil content of the *AtOLE3*-OE seeds was the highest, reaching 35.8%, an increase of 10.8% compared with the WT. The average oil content also increased to a similar extent in *AtOLE1*-OE (34.7%), *AtOLE3*-OE (35.0%), *BnaOLE1*-OE (33.3%), *BnaOLE2*-OE (33.3%) and *BnaOLE4*-OE (34.8%). On the other hand, the oil content in the *BnaOLE3*-OE seeds decreased to a value of only 29.9%.

**Table 3:** Relative content of three C18 fatty acids of transgenic seeds

	*AtOLE1*-OE	*AtOLE2*-OE	*AtOLE3*-OE	*BnOLE1*-OE	*BnOLE2*-OE	*BnOLE3*-OE	*BnOLE4*-OE	WT
C18:1	17.77124	16.30847	16.34432	16.85946	16.26213	16.99048	15.48557	16.36811
C18:2	31.1659	30.47247	30.11299	31.50722	31.20111	30.65505	29.96506	29.40841
C18:3	16.68444	17.51728	17.55592	16.67468	16.76694	16.61466	17.91633	17.8015

The fatty acid profile also changed, especially for the 18C unsaturated fatty acids. The levels of all three of the main 18C unsaturated fatty acids in the seeds of the OLE lines increased or decreased to different degrees. In addition to *BnaOLE4*, the transgenic lines showed increased oleic and linoleic acid levels; however, the relative level of linolenic acid decreased. In addition, the eicosaenoic acid content also showed a decreasing trend. Among all of the transgenic lines, the fatty acid profile of *AtOLE1*-OE seeds showed the maximum variation. The oleic acid content increased from 15.8 to 17.2%, and the linoleic acid content increased from 29.5 to 32.6%, accompanied by decreased linolenic acid (from 17.8 to 15.3%) and eicosaenoic acid (from 20.1 to 17.8%) levels. *BnaOLE1* exhibited the largest increase in linoleic acid content (7.1%), followed by *BnaOLE2* (6.1%) and *BnaOLE3* (6.0%). As a consequence, the linolenic acid and eicosaenoic acid levels of these three transgenic lines decreased more than those of the other transgenic lines.

In addition to lipid metabolism, seed weight was also affected in the transgenic lines **(**Fig. 7c), and it was revealed that the thousand-seed weight (TSW) in all of the transgenic lines increased to different degrees. *BnaOLE1*-OEs had the largest average TSW, which was 24.4% higher than that of the WT. Considering the seed oil content and the TSWs of the transgenic lines, it was concluded that overexpression of oleosin could increase the total fatty acid production per seed. The linoleic acid content can reach 1.98 mg per thousand seeds in the *AtOLE1*-OE lines, while the level in the control is only 1.44 mg, representing a 34.7% increase in the *AtOLE1*-OE lines, more than that in the other transgenic lines. The oleic acid content per thousand seeds in the transgenic lines also showed a similar increase as the linoleic acid content. Moreover, the linolenic acid and eicosaenoic acid levels showed a slight increase compared with the levels in the WT. It was revealed that *AtOLE1*-OE contains 1.03 mg of linolenic acid and 1.12 mg of eicosaenoic acid per thousand seeds, while the WT seeds contain 0.9 mg linolenic acid and 0.99 mg eicosaenoic acid. In a word, overexpression of *OLE* might affect the relative content of fatty acids, but could enhancing the linoleic acid content without decreasing the yield of other fatty acids.

### Overexpression of *OLE*s in *Arabidopsis* could affect oil body size

**Fig. 8:**
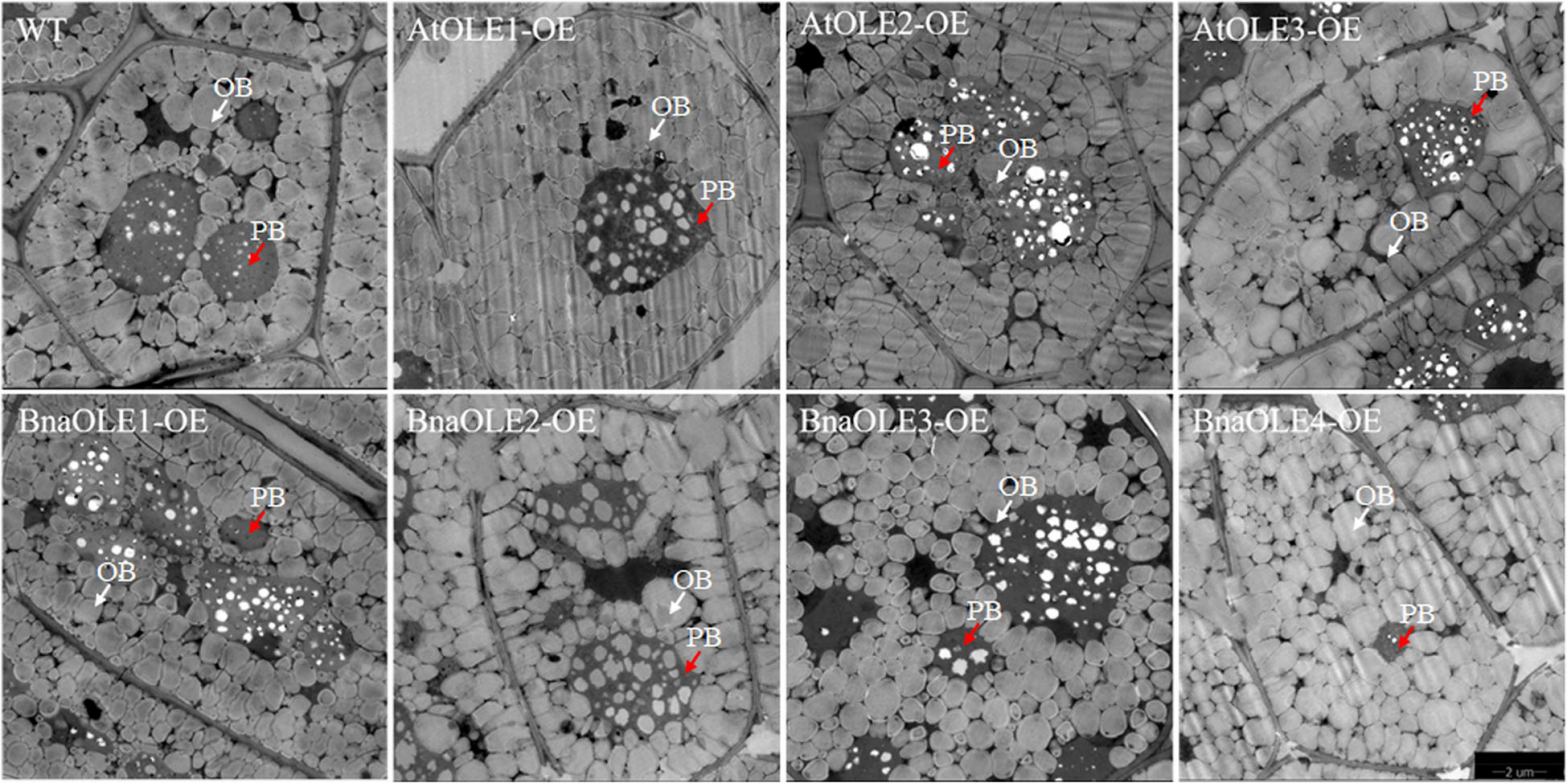
TEM images of oil bodies in cotyledons of transgenic seeds. (Bar = 2 μm). WT represents the cotyledons of wild-type seeds; AtOLE1-OE, AtOLE2-OE, AtOLE3-OE, BnOLE1-OE, BnOLE2-OE, BnOLE3-OE and BnOLE4-OE represent the cotyledons of transgenic seeds overexpressing *AtOLE1*, *AtOLE2*, *AtOLE3, BnOLE1, BnOLE2, BnOLE3* and *BnOLE4,* respectively. OB indicates oil bodies, and PB indicates protein bodies

**Fig. 9:**
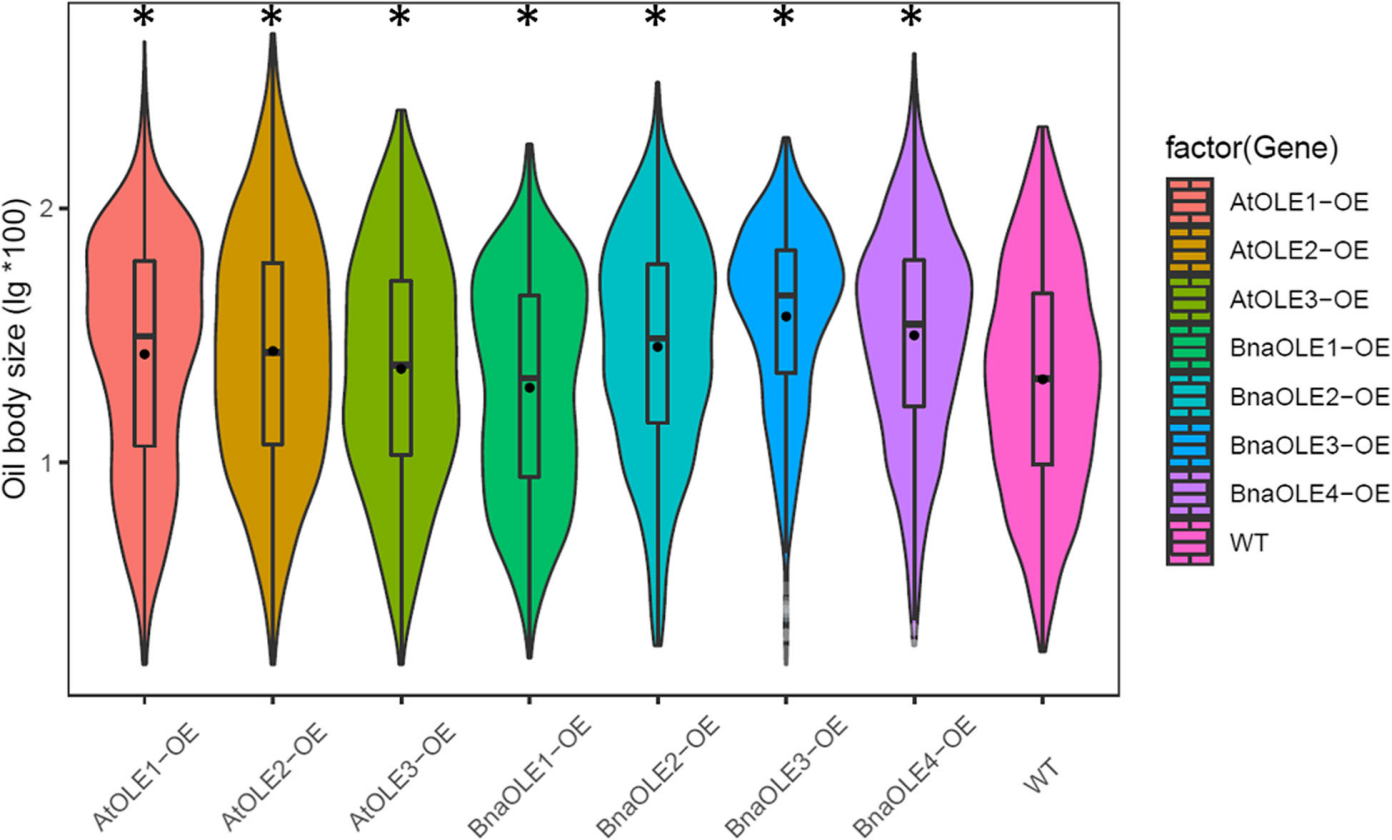
Distribution of different sizes of oil bodies in transgenic seeds. The Y-axis represents the logarithm of oil body area to the base 10 of the concentration. Different colors represent different transgenic lines. T-test was used to measure significance differences between lines and the transgenic lines showed significant difference with the control (* represent that *P* < 0.05)

The oil body size in the seeds of transgenic lines was also analyzed. The average oil body size of most of the transgenic lines was significantly higher than that of the WT, except for *BnaOLE1*-OEs (*P* < 0.01) (Fig. 8, Fig. 9). The average oil body size of *BnaOLE1*-OEs was only 0.29 μm^2^, whereas the average oil body size of the WT was 0.33 μm^2^. The average oil body size of all six transgenic lines ranged from 0.41 to 0.48 μm^2^, representing a 24 to 45% increase compared to the control. Further analysis revealed that the largest oil body was observed in *AtOLE2*-OEs, reaching 4.842 μm^2^, more than two times larger than the largest oil body in WT seeds (2.08 μm^2^). Considering the different oil body size distributions, in cotyledons of *BnaOLE1*-OEs, the percentage of oil bodies with sizes less than 0.6 μm^2^ was similar to that in the WT, whereas the number of oil bodies with sizes less than 0.9 μm^2^ was greater than that in the WT. Taken together, these results showed that overexpression of *BnaOLE1* resulted in relatively few large oil bodies, which led to a relatively small average oil body size (Additional file 4: Figure S4).

### Overexpression of *OLE* genes could affect freezing tolerance

**Fig. 10:**
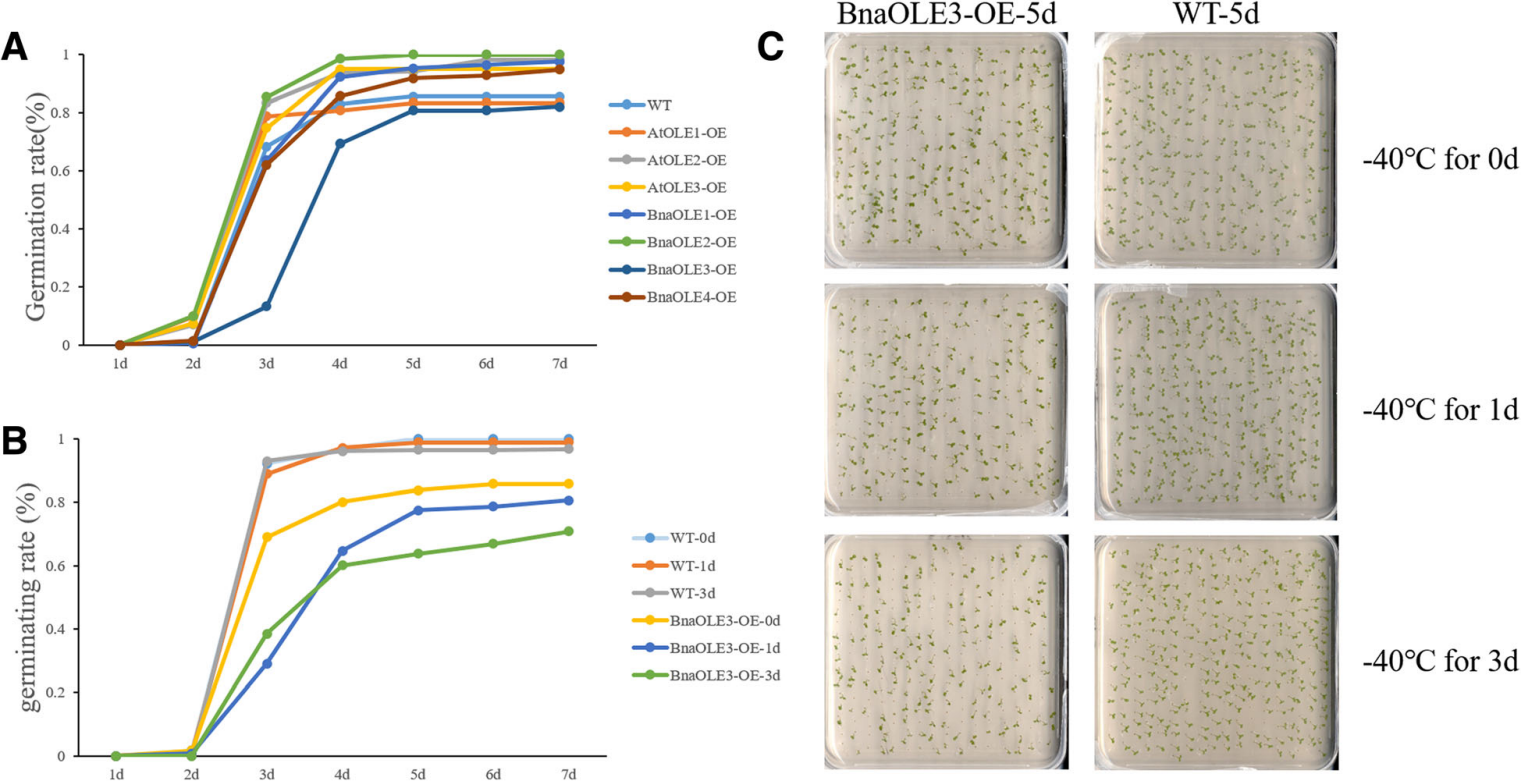
Germination rate of *Arabidopsis* seeds after freezing treatment. **a** Germination rate of *Arabidopsis* seeds after freezing treatment for 24 h. **b** Germination rate of *Arabidopsis* seeds overexpressing *BnaOLE3* and WT seeds after freezing treatment for different durations. T-test was used to measure significance differences between lines and the transgenic lines showed significant difference with the control. **c** Germination rate of *Arabidopsis* seeds overexpressing *BnaOLE3* and WT seeds after freezing treatment for different durations 5 days after sowing

A previous study showed that oleosin might increase the resistance of seeds to freezing, so we asked whether the evaluated oleosin level could reduce the injuries caused by freezing treatment. The transgenic and WT seeds were treated by freezing at − 40 °C for 1 day, followed by sowing on MS medium containing 1% agar. Surprisingly, the freezing treatment did not affect seed germination, except for seeds expressing *BnaOLE3* (Fig. 10). Three days after sowing, the germination rate of seeds expressing *BnaOLE3* was lower than that of other seeds. Further analysis showed that prolonged freezing time could harm seed germination severely. After treatment with low temperature for 3 days, the germination rate decreased from 85.7 to 70%. These results show that heterologous expression of *BnaOLE3* could reduce cold resistance.

## Discussion

Plant seeds that store lipids in oil bodies are used for seed germination and other physiological and biochemical metabolic processes. The oil body consists of TAG, a phospholipid monolayer enclosing the neutral lipids and oleosins inserted into the half-unit membrane [5, 7]. From primitive green algae to higher plants, oleosin is widely distributed in different species [21], indicating the important roles of oleosin in oil body biogenesis and stabilization. Due to the high conservation of oleosin, many studies have focused on the evolutionary relationships of oleosins [21, 23]. It was revealed that there are six lineages of oleosins (P, U, SL, SH, T and M) in plants; some lineages exist in only a single family, such as T oleosin and M oleosin, which are found in only *Lauraceae* and *Brassicaceae*, respectively. The gene evolution of oleosins has been reviewed for some species; however, the identification and annotation of oleosins in *B. napus*, an important oil crop worldwide, has received little attention. In the present study, 48 oleosin genes from the *B. napus* genome were identified based on the conserved domain, and these genes were divided into four lineages of oleosins (U, SL, SH, and T). We did not detect P oleosins and M oleosins in *B. napus*; these lineages were only found in algae and *Lauraceae,* respectively. Nearly half of the identified oleosins were T oleosins, and the other three lineages were present at similar levels. A previous study has shown that most oleosins possess no introns [23]. *Liu* et al [38] hypothesized that introns of oleosins were gained early in evolution and then lost, so most of oleosins possess no introns. The present results revealed the presence of at least one intron in the oleosin sequences, except U oleosins, in *B. napus*. Which was similar with the previous report that there are few introns in the oleosin genes of other species except for Brassicaceae plants [16]. And in Brassicaceae plants, most of SL, SH and T oleosins contain introns and U oleosins contain few oleosins. Although SH oleosins and SL oleosins evolved from the U oleosins [5], there is a difference in intron number between these oleosins; SH oleosins have more introns than SL oleosins. Based on the genomic structures of oleosins, *BnaOLE23* and *BnaOLE35* were the two exceptions among U oleosins, each possessing six introns; however, the homologous genes in *Arabidopsis AtOLE5* had no intronic structure. These two genes contain more nucleotides than other oleosin genes and it might indicate that the increased gene length during evolution lead to the more complex gene structures. Although the intron structures and sequences differed, all of the introns in the oleosins were U2-type splice GT-AG introns [33, 34].

Gene duplication increased the gene number and led to functional differentiation of genes for adaptation to the environment during evolution. Many studies have revealed that *Brassica* species have undergone whole-genome duplication (WGD) events during evolution. In addition, several independent lineage-specific WGD events have been identified in *Brassicaceae* [35, 39]. For the WGT event in *Brassica* species, the *BnaOLE* genes experienced gene duplication events. Genetic structure and phylogenetic analyses showed that *BnaOLE*s share a close phylogenetic relationship and similar structure with corresponding homologous genes in three other *Brassicaceae* plants. The results were also further confirmed by the *Ka/Ks* ratios (less than one), suggesting that the oleosin genes in *Brassicaceae* plants had experienced purifying selection (Additional file 6: Table S2). The tandemly duplicated genes were also detected in the *BnaOLE* gene family, and all twelve tandemly duplicated genes belonged to T oleosins. One underlying reason might be that T oleosins formed gene clusters in the *Arabidopsis* genome. Gene duplication played an important role in the genetic evolution of this locus; however, redundant gene numbers led to relatively low conservation because the *Ka/Ks* ratio of T oleosins was relatively higher than that of the other three lineages.

Oleosin was first identified in maize in 1990 [15], and previous studies have shown that oleosin, the main structural protein of the oil body, can keep oil droplets stable and prevent oil body aggregation during seed dehydration [40]. However, the role of oleosin in the storage and activation of lipids in seeds and other cellular activities remains unclear. Oleosins play an important role in the formation of oil droplets and accumulation of lipids. Reduction in oleosin content in rice seeds can lead to decreased oil content and increased oil body size in seeds [26]. However, few studies have investigated the relationship between the oleosin protein content and oil body size. In the present study, three *Arabidopsis* oleosin genes and their homologs from *B. napus* were selected for verification of their effect on seed oil content in *Arabidopsis*. Homozygous T3 transgenic seeds were used for all further analyses. The cotyledons of the seeds were used for transmission electron microscopy (TEM) analysis; the seed oil body sizes of the transgenic lines were significantly higher than those of the WT, except for *BnaOLE1* (*P* < 0.01). *BnaOLE1* overexpression resulted in a significant decrease in oil body size compared with that in WT seeds (*P* < 0.01), with an average size of 0.292 to 0.332 μm^2^, which is consistent with the previous hypothesis that *AtOLE1* might restrict oil body size. Considering the different oil body size distributions, similar to the overall result, there were more small oil bodies in transgenic seeds than in WT seeds. In the *BnaOLE1*-OE lines, the percentage of oil bodies with sizes less than 0.9 μm^2^ was higher than that in the WT, leading to a small oil body size overall. In contrast, the percentage of oil bodies in other transgenic seeds was higher than that in the WT, and the largest oil body, which was two times larger than the largest oil body in the WT, was observed in *AtOLE2* seeds. These results indicate that abnormal gene expression could disrupt the natural oil body formation process, which could result in the emergence of a relatively large oil body, even an unusually large oil body, instead of a simple negative correlation between oil body size and oleosin content.

After knocking out the *Arabidopsis* oil body protein gene, the oleic acid (C18:1) content in seeds decreased, and the eicosenoic acid (C20:1) content increased [29]. On the other hand, overexpression of castor oleosin in *A. thaliana* can greatly increase the accumulation of hydroxyl fatty acids in seeds [29], and coexpression of oleosin with other important lipid synthesis-related genes, such as *DGAT, FADX* and *WRI1,* could further improve target production [41–44]. For example, coexpression of *ScDGA1* and *AtOLE3* resulted in a nearly 50% increase in oil content compared with transformants that only expressed the single gene *ScDGA1* [42]. In the present study, the linoleic acid (C18:2) content in transgenic seeds was higher than that in the control, and the eicosaenoic acid (C20:1) content was lower than that in the WT seeds, which was opposite trend compared to that in the *ole* mutant seeds reported previously [45]. This result could be partly explained by the fact that oleosin can be coexpressed with *FAD2* and *FAD3* in *Arabidopsis* [46, 47]; therefore, the expression of oleosin could affect the levels of the substrate and product of the two related enzymes. Previous research has shown that overexpression of oleosin from *Carthamus tinctorius* L*.* and soybean could alter the seed oil content [48, 49]. Although in the present study, the oil content of the transgenic lines showed different variation trends, the highest oil content reached 37.9%, which was 14.8% higher than that in the WT. Lipids are the main form of storage in *Arabidopsis* seeds, and changes in oil content may affect seed quality. Alteration of the expression of lipid metabolism-related genes such as *DGAT*, *FAX1* and *WRI1* affects oil content and seed weight [50–52]. The most important phenomenon observed in the present study was that the TSWs of the transgenic lines increased. Taken together, these results showed that overexpression of *OLE*s could increase oil production and the linoleic acid yield. For example, the linoleic acid content in *AtOLE1*-OE seeds reached 2.37 mg per thousand seeds, while the value was only 1.46 mg for the control. The highest oil output per thousand seeds of the transgenic lines reached 7.8 mg, which was 57% higher than the average yield of the WT. These results suggested that increased oleosin content might promote lipid synthesis.

## Conclusion

In this study, we identified 48 oleosin genes in *B. napus* and divided these genes into four types, and at least one intron was found in all the oleosin sequences except U oleosins. Over half of the *BnaOLE* genes are associated with segmental duplications. Synteny analysis showed that most of the oleosin genes in *B. napus* are relatively conserved, and all of them were faced purifying selection pressure in evolution. In addition, several important oleosins from *Arabidopsis* and *B. napus* were cloned, and the functions of these oleosins in *Arabidopsis* seeds were analyzed. It was shown that overexpression of oleosin genes in *A. thaliana* has a weak effect on seed oil content but could increase the linoleic acid content (13.3% at most) and decrease the peanut acid content (11% at most) compared to the levels in the WT. The TSWs also increased, which could lead to increased total lipid production. The oil body size in transgenic seeds was larger than that in the WT, except for *BnaOLE1*. This study provides a foundation for future studies regarding the oleosin superfamily in *B. napus*.

## Methods

### Identification of oleosin genes in *B. napus* and other related species

*Arabidopsis* genes were searched in the TAIR and Phytozome databases [53] using oleosin as the key word query and then confirmed using the hidden Markov model (HMM) with the Pfam [54], SMART [55] and NCBI Conserved Domain Search databases [56]. The oleosin genes were identified in *B. napus* based on homologous sequences from *A. thaliana* using the BLAST search program in the CNS-Genoscope database [57]. The oleosin gene sequences from *B. rapa* and *B. oleracea* were obtained from the BRAD database [58, 59]. The oleosin sequences from *B. napus*, *B. rapa*, and *B. oleracea* were also confirmed in the Pfam, SMART and NCBI Conserved Domain Search databases. Redundant sequences and some proteins containing no characteristic oleosin domain were removed manually. According to the gene synteny with oleosins of *Arabidopsis* and the corresponding chromosomal distribution, a univocal name consisting of two italic letters indicating the source organism and the family was assigned to each oleosin gene **(**Additional file 5: Table S1**)**. When searching the sequence information, the number of amino acids, coding sequence (CDS) lengths, intron and exon numbers, and chromosomal locations of the *Bna*OLE genes were obtained from the CNS-Genoscope and BRAD databases. ExPASy was used to calculate the physicochemical parameters, including the molecular weight (kDa), pI and GRAVY, of all the identified oleosin proteins [60].

### Gene structure, multiple sequence alignment and phylogenetic analysis of oleosin genes

After obtaining complete oleosin CDS and genomic sequences from the CNS-Genoscope, TAIR and BRAD databases, GSDS [61] was used to illustrate the exon-intron structures of the oleosin genes and the intron phases. If an intron is between two complete codons, then the intron phase was defined as 0; if an intron is after the first or second nucleotide within the codon, the intron phase was defined as phase 1 or phase 2, respectively. Multiple sequence alignment of the oleosin protein sequences of *A. thaliana*, *B. rapa*, *B. oleracea* and *B. napus* was performed using ClustalW software [62]. Then, the conserved central domain was used to construct an unrooted cladogram using MEGA 6 with the neighbor-joining (NJ) method, and a bootstrap analysis was conducted using 1000 replicates [63]. These conserved portions were also predicted by MEME to show the highly conserved amino acids of oleosin genes [64].

### Chromosomal location and gene duplication of the *Bna*OLE family genes

The chromosomal locations of the *Bna*OLE genes from the *B. napus* database and the syntenic relationships between the *Bna*OLEs and *At*OLEs, *Bra*OLEs, and *Bol*OLEs were acquired with the syntenic genes search tool in the BRAD database. Then, Circos was used to show the oleosin distribution in the *B. napus* genome and the syntenic relationships in the four types of plants [65]. TBtools was used to calculate the nonsynonymous and synonymous substitution rates of every paralogous pair of oleosin genes between *A. thaliana* and *B. napus*. The *Ka/Ks* ratio represents the ratio of the number of nonsynonymous substitutions per nonsynonymous site (*Ka*) to the number of synonymous substitutions per synonymous site (*Ks*). When the *Ka/Ks* ratio was less than 1, purifying selection occurred, and otherwise, positive selection occurred. When two genes with sequence distances less than 50 kb belong to the same gene family, we defined these genes as tandemly duplicated genes.

### Gene cloning, vector construction and gene transformation

All plant materials and plant expression vectors used in this study were stored in the laboratory of Prof. Li Maoteng of Huazhong University of Science and Technology. Arabidopsis was cultured at 21 ± 2 °C with a 16 h light/8 h dark photoperiod at a relative humidity of 60–90%. *B. napus* cultivar was grown in the experimental field of the Huazhong Agricultural University (30°36′ N, 104°18′ E), Wuhan, China. First, the primers used to clone the OLE genes were designed using Oligo 6.0 based on the gene sequence obtained from the TAIR and CNS-Genoscope databases, and restriction sites were added to the primers. The RNAprep Pure Plant Kit (TIANGEN BIOTECH (BEIJING) CO., LTD) was used to isolate total RNA from the developing siliques of *A. thaliana* and *B. napus*, and the PrimeScript™ RT Master Mix Kit (TaKaRa) was used to synthesize first-strand DNA according to the manufacturer’s instructions. This DNA was used as a template for subsequent PCR amplification. The target genes were integrated into the plant expression vector using the In-Fusion® HD Cloning Kit (Clontech). All vectors used in this study contained *DsRED*, a type of fluorescent protein that can emit red light under excitation by green light.

The recombinant vector was transformed into *Col Arabidopsis* by the floral dip method by using *Agrobacterium tumefaciens*. We could identify the transgenic seeds by observing the red light with the help of a filter. In addition, T3-generation transgenic *Arabidopsis* was used to investigate plant phenotypes. Twenty days after flowering, the siliques of transgenic *Arabidopsis* were harvested to analyze the expression of target genes. SYBR Green Real-time PCR Master Mix was used to perform RT-qPCR.

### Measurement of oil content and fatty acid compositions

The seeds were first weighed using a semi-microanalytical balance and then placed on a glass plate. The seeds were scattered such that there was no overlap among seeds for further analysis. Then, the Seed Count image analysis system (WSeen Detection of Zhejiang Sci-tech University, Hangzhou, Zhejiang, China) was used to scan the seeds and analyze the seed number, seed area, seed length and seed width; the TSW was also determined.

The method for oil content measurement has been previously reported, which we used with some modification [66]. Five milligrams of dry seeds were used for oil content and fatty acid analysis. After placing the seeds into a glass tube, 1 ml of 2.5% sulfuric acid-methanol solution, 0.4 ml of toluene and 0.2 ml of a 2 mg/ml C17:0 solution in toluene were added. The mixture was vortexed and then heated in a 90 °C water bath for 1 h, and then, 1.8 ml of ddH_2_O and 1 ml of hexane were added after the tube had cooled. After 15 min, the supernatant was filtered using a 0.45-μm microporous membrane. The filtrate was used to determine the fatty acid content by GC using an Agilent 7890A instrument. At least three replicate samples were examined for all of the experiments.

### Measurement of oil body sizes

The transgenic seeds that emitted red light under excitation by green light were selected for TEM analysis. The dry seeds were first fixed using 2.5% glutaraldehyde solution, and then the manually isolated cotyledons were sliced and photographed. All the associated procedures have been described previously [11], and all the TEM imaging was completed by the Wuhan Institute of Virology. At least three samples were used to measure oil body size. At least 3 TEM images of the cotyledons of each transgenic line were used to measure oil body size using ImageJ. T-test was used to measure significance differences between lines.

### Freezing treatment

The method for the freezing treatment has been previously reported by Shimada et al. [6], which we used with some modifications. The dry *Arabidopsis* seeds were treated by freezing at − 40 °C for 1 or 3 days. Then, the dry seeds were first washed with 70% ethanol for 1 min and then washed 3 times with ddH_2_O. Fifty percent disinfectant 84 was used to further eliminate bacteria, and the samples were then washed with ddH_2_O 3 times. Then, the seeds were sown on MS medium containing 1% agar. We took a picture of the dish every 24 h and determined the germination rate.

## Additional files

Additional file 1: **Figure S1** Sequence alignment and MEME analysis of *B. napus* oleosin genes. (A) MEME logo of the conserved Pro knot; (B) sequence alignment of the 72 conserved amino acids in 48 *B. napus* oleosin proteins. The relatively highly conserved amino acids are marked in different colors. (PNG 3427 kb)

Additional file 2: **Figure S2** Genetic structure of *A. thaliana, B. oleracea, B. rapa* and *B. napus* oleosin genes**.** The motifs in oleosin are shown on the left, and the genetic structure is shown on the right. The numbers near the introns indicate the intron phase. (PNG 579 kb)

Additional file 3: **Figure S3**. Subcellular localization of AtOLE1, AtOLE2, AtOLE3 and AtOLE4. A, B, C, and D represent AtOLE1; E, F, G, and H represent AtOLE2; I, J, K, and L represent AtOLE3; and M, N, O, and P represent AtOLE4. A, E, I, M: EGFP fluorescence; B, F, G, N: chloroplast autofluorescence; C, G, K, N: background; D, H, L, P: merged EGFP and chloroplast fluorescence. (PNG 3259 kb)

Additional file 4: **Figure S4** Distribution of different sizes of oil bodies in transgenic seeds. Different sizes of oil bodies are marked with different colors. (TIF 12720 kb)

Additional file 5: **Table S1** Gene names and the corresponding gene IDs. (XLS 34 kb)

Additional file 6: **Table S2** Ka/Ks ratios between all of the oleosins from *A. thaliana*, *B. oleracea*, *B. rapa*, and *B. napus*. (XLS 1511 kb)

## Data Availability

The datasets generated and analyzed during the present study are available from the corresponding author on reasonable request.

## Abbreviations

CDS: Coding sequence
DGAT: Diacylgycerol acyltransferase
HMM: Hidden Markov model
MEME: Multiple Expectation Maximization for Motif Elicitation
OBAP1: Oil body-associated protein 1
QTL: Quantitative trait locus
TAG: Triacylglycerol
TAIR: the Arabidopsis Information Resource
TEM: Transmission electron microscopy
TSW: Thousand-seed weight
WGD: Whole-genome duplication
WT: Wild type
