# Supplementary figures and images for: Genome-wide identification and functional analysis of oleosin genes in Brassica napus L
Source: BMC Plant Biol. 2019 Jul 4;19:294. doi: 10.1186/s12870-019-1891-y (PMC6610931; doi:10.1186/s12870-019-1891-y)

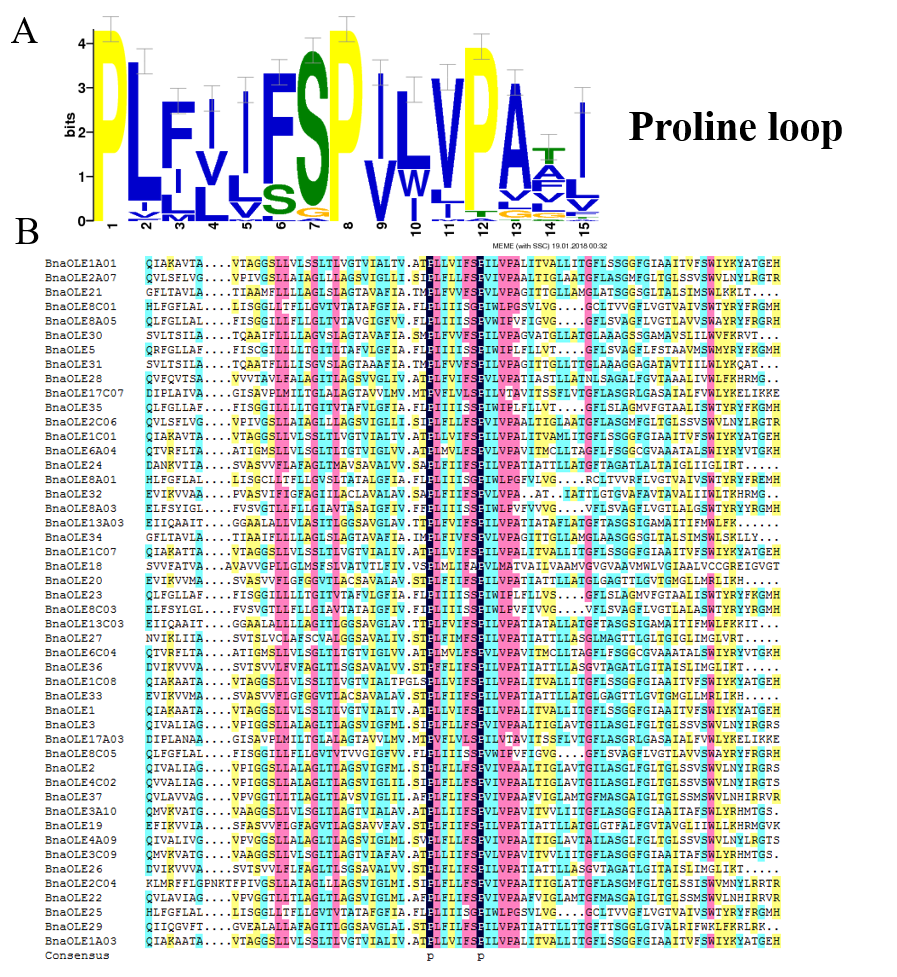

Supplement: Supplementary file 1 — Figure S1 Sequence alignment and MEME analysis of B. napus oleosin genes. (A) MEME logo of the conserved Pro knot; (B) sequence alignment of the 72 conserved amino acids in 48 B. napus oleosin proteins. The relatively highly conserved amino acids are marked in different colors. (PNG 3427 kb) [file 12870_2019_1891_MOESM1_ESM.png]

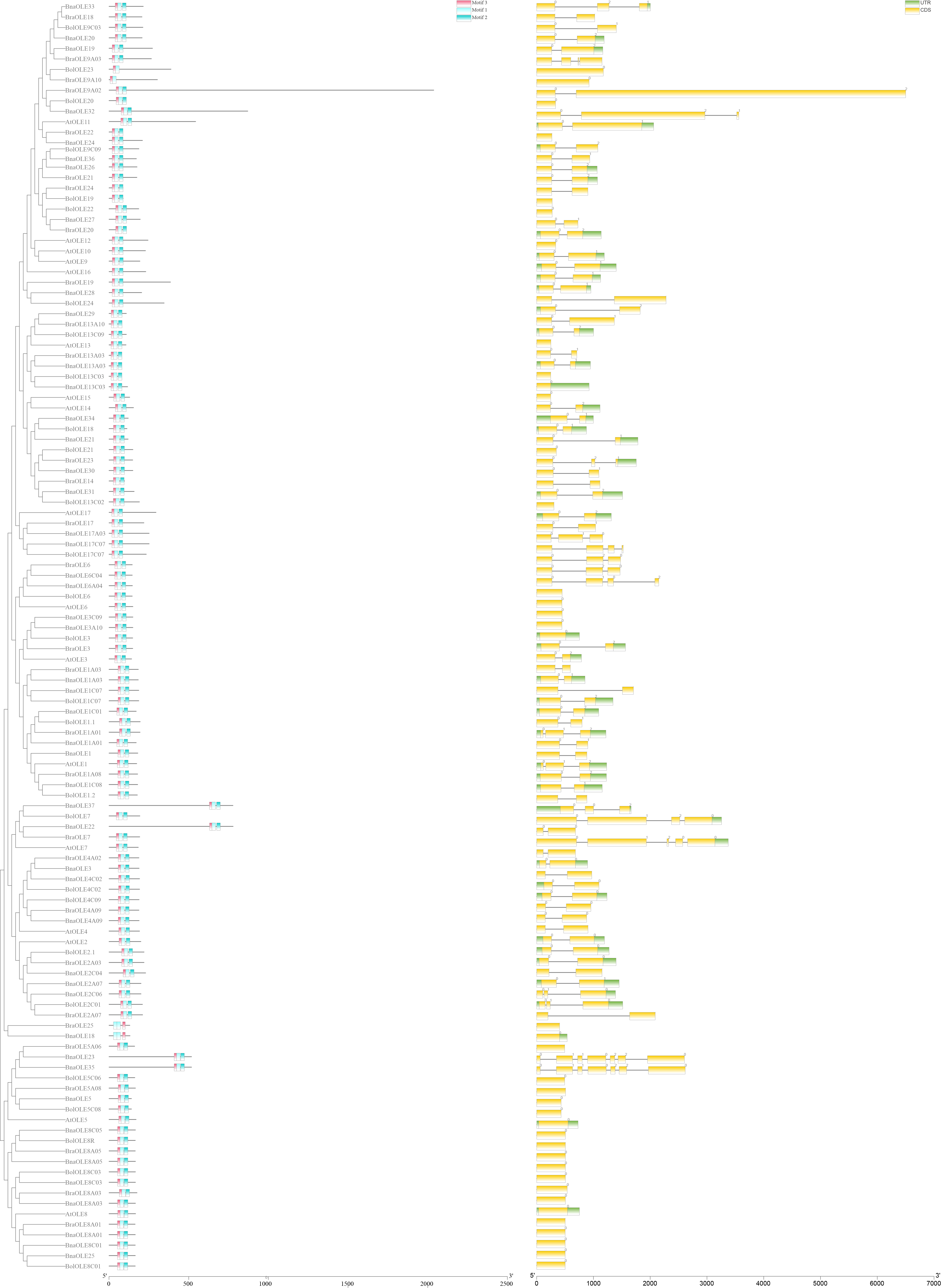

Supplement: Supplementary file 2 — Figure S2 Genetic structure of A. thaliana, B. oleracea, B. rapa and B. napus oleosin genes. The motifs in oleosin are shown on the left, and the genetic structure is shown on the right. The numbers near the introns indicate the intron phase. (PNG 579 kb) [file 12870_2019_1891_MOESM2_ESM.png]

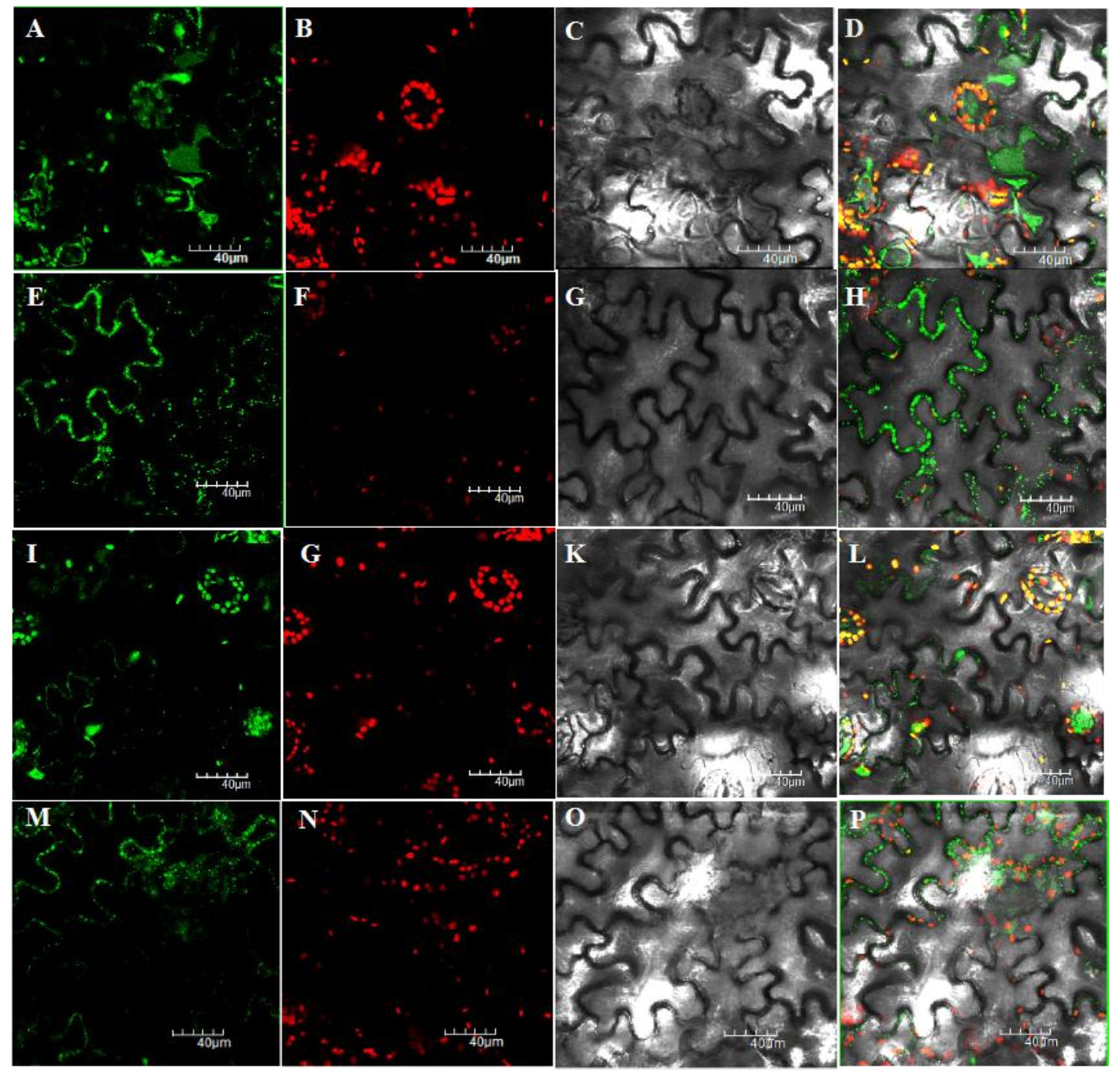

Supplement: Supplementary file 3 — Figure S3. Subcellular localization of AtOLE1, AtOLE2, AtOLE3 and AtOLE4. A, B, C, and D represent AtOLE1; E, F, G, and H represent AtOLE2; I, J, K, and L represent AtOLE3; and M, N, O, and P represent AtOLE4. A, E, I, M: EGFP fluorescence; B, F, G, N: chloroplast autofluorescence; C, G, K, N: background; D, H, L, P: merged EGFP and chloroplast fluorescence. (PNG 3259 kb) [file 12870_2019_1891_MOESM3_ESM.png]

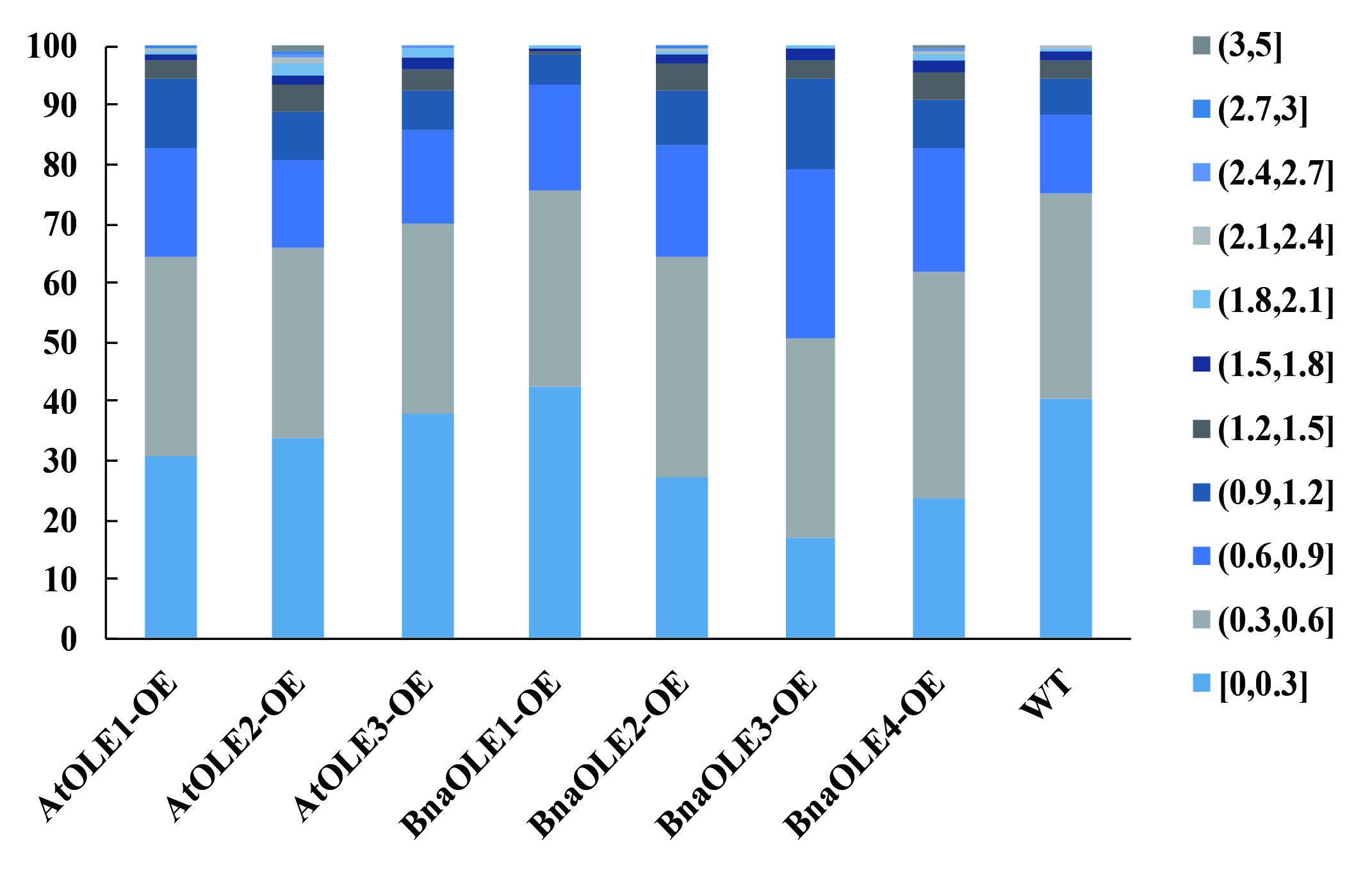

Supplement: Supplementary file 4 — Figure S4 Distribution of different sizes of oil bodies in transgenic seeds. Different sizes of oil bodies are marked with different colors. (TIF 12720 kb) [file 12870_2019_1891_MOESM4_ESM.tif]
